# A high-throughput sequencing assay to comprehensively detect and characterize unicellular eukaryotes and helminths from biological and environmental samples

**DOI:** 10.1186/s40168-018-0581-6

**Published:** 2018-10-29

**Authors:** Matthew V. Cannon, Haikel Bogale, Lindsay Rutt, Michael Humphrys, Poonum Korpe, Priya Duggal, Jacques Ravel, David Serre

**Affiliations:** 1 Institute for Genome Sciences, University of Maryland School of Medicine, Baltimore, MD USA; 2 Department of Epidemiology, Johns Hopkins School of Public Health, Baltimore, MD USA

**Keywords:** Eukaryotic pathogens, rRNA sequencing, Microbiome, Infectious diseases, Food and water safety, High-throughput screening

## Abstract

**Background:**

Several of the most devastating human diseases are caused by eukaryotic parasites transmitted by arthropod vectors or through food and water contamination. These pathogens only represent a fraction of all unicellular eukaryotes and helminths that are present in the environment and many uncharacterized organisms might have subtle but pervasive effects on health, including by modifying the microbiome where they reside. Unfortunately, while we have modern molecular tools to characterize bacterial and, to a lesser extent, fungal communities, we lack suitable methods to comprehensively investigate and characterize most unicellular eukaryotes and helminths: the detection of these organisms often relies on microscopy that cannot differentiate related organisms, while molecular assays can only detect the pathogens specifically tested.

**Results:**

Here, we describe a novel sequencing-based assay, akin to bacterial 16S rRNA sequencing, that enables high-throughput detection and characterization of a wide range of unicellular eukaryotes and helminths, including those from taxonomical groups containing all common human parasites. We designed and evaluated taxon-specific PCR primer pairs that selectively amplify all species from eight taxonomical groups (Apicomplexa, Amoeba, Diplomonadida, Kinetoplastida, Parabasalia, Nematoda, Platyhelminthes, and Microsporidia). We then used these primers to screen DNA extracted from clinical, biological, and environmental samples, and after next-generation sequencing, identified both known and previously undescribed organisms from most taxa targeted.

**Conclusions:**

This novel high-throughput assay enables comprehensive detection and identification of eukaryotic parasites and related organisms, from a wide range of complex biological and environmental samples. This approach can be easily deployed to many settings and will efficiently complement existing methods and provide a holistic perspective on the microbiome.

**Electronic supplementary material:**

The online version of this article (10.1186/s40168-018-0581-6) contains supplementary material, which is available to authorized users.

## Background

Eukaryotic parasites are directly responsible for over one million human deaths yearly, causing widespread infectious diseases, such as malaria [1] or infant diarrheas [2, 3], and many of the neglected tropical diseases [4, 5]. This highly heterogeneous group of organisms that includes many different types of unicellular eukaryotes and various helminths also threatens food and water safety [6–9] and can cause livestock epidemics of dramatic economic consequences [10–13].

Beyond this direct impact of health, unicellular eukaryotes and helminths can also have subtler consequences by modifying their environment and dysregulating the microbiome. For example, several studies have shown that *Giardia* [14–16], *Entamoeba* [17, 18], and helminths [19–21] can alter an individual’s gut microbiome [15, 22, 23]. Alternatively, these organisms can sometimes positively influence the microbiome and an individual’s health [24–28]. Recent studies of more than 9000 infants with diarrhea, and their matched controls, have also shown that most individuals carried several “pathogens,” regardless of their disease status and that no simple correlation existed between any given organism and disease [29, 30]. These studies emphasize the complex interactions occurring between microorganisms and the need to comprehensively characterize all organisms present in the microbiome to better understand its regulation. Unfortunately, such exhaustive studies are difficult to implement as, in striking contrast to the situation in bacteria [31], we still lack efficient tools to comprehensively detect and identify unicellular eukaryotes and helminths. Many diagnostics still rely on labor-intensive microscopic analyses, and molecular approaches are often limited to testing for proteins or nucleic acids from a few specific pathogens. These approaches can not only miss important parasites that are not directly tested, but also lead to misclassifications and agglomeration of organisms with very different phenotypes. Several studies have investigated using universal eukaryote primers combined with next-generation sequencing to survey parasites [32–36]. However, this approach has limitations that reduce its effectiveness. First, eukaryotic primers can, by design, amplify a wide range of organisms and these unwanted DNA molecules might completely overwhelm the signal from minute amount of parasites. This lack of specificity would, for example, significantly hamper studies of human biological samples, arthropod disease vectors, or food safety. Second, even if “contaminating” DNA is not an issue, the use of a single, generic, primer pair may fail to recapitulate the diversity present in one sample: the DNA from one species could swamp the signal from other taxa represented by fewer DNA molecules or less efficiently amplified.

Here, we describe a novel sequencing-based assay that enables high-throughput, targeted screening of complex biological and environmental samples and detection and characterization of most eukaryotic parasites and related organisms, including all Apicomplexans, Amoebas, Diplomonads, Kinetoplastids, Parabasalids, Nematodes, Platyhelminthes, and Microsporidians. We show that this assay can efficiently identify known pathogens as well as organisms that have not yet been characterized and demonstrate the potential of this approach to significantly improve microbiome, clinical, agricultural, environmental, or food safety studies.

## Results

### Design and assessment of PCR primers to amplify most eukaryotic parasites

We designed PCR primers to amplify a wide range of unicellular eukaryotes and helminths, including all common human parasites, using the same concept as used for bacterial 16S ribosomal RNA (rRNA) gene sequencing. Briefly, each primer pair was designed to (i) amplify all members of the selected taxon, (ii) not amplify DNA from other organisms (including bacteria, mammals, and arthropods), (iii) amplify a DNA sequence carrying sufficient genetic information to enable differentiating among species, and (iv) generate a short enough DNA fragment to be sequenced on a high-output DNA sequencer, with sufficient read overlap for error correction. In practice, the availability of sufficient annotated DNA sequences also restricted our selection to 18S rRNA genes.

Overall, we designed 13 primer pairs that, in silico, amplify DNA from most Apicomplexans, Amoebas, Diplomonads, Kinetoplastids, Parabasalids, Nematodes, Platyhelminthes, Microsporidians, and *Blastocystis* (Table 1, Additional file 1: Table S1) and should provide a comprehensive perspective on unicellular eukaryotes and helminths able to infect humans and other mammals [37]. In some cases, design of several complementary primer pairs was necessary to efficiently amplify highly diverse taxa while avoiding off-target amplification of mammalian and insect DNA (Additional file 1: Figure S1).

**Table 1:** Summary of the primer characteristics and specificity

Taxon targeted	Amplicon length (in bp)	No. species amplifiable (%NCBI*)	Matching single genus (mean)	Matching single species (mean)	Specificity (%on target)	Forward primer	Reverse primer
Amoebozoa	333–420	265 (69.9%)	98.7% (1)	83.7% (1.3)	47.4%	GAATTGACGGAAGGGCACAC	GCCCYRTCTAAGGGCATCAC
Apicomplexa A	244–248	434 (80.4%)	84.8% (1.3)	55.9% (6.8)	99.1%	GACCTATCAGCTTTCGACGG	CCCTCCAATTGWTACTCTGGR
Apicomplexa B	228–244		95.6% (1.2)	73.1% (3.4)	94.1%	TGYGTTTGAATACTAYAGCATGG	TCTGATCGTCTTCACTCCCTT
Apicomplexa C	420–470		99.1% (1)	76.2% (1.4)	83.1%	TTGGMCTACCGTGGCARTGA	TCAAGGCAAHWGCCTGCTT
Blastocystis	303–307	4 (66.7%)	100% (1)	94.1% (1.1)	80.0%	TGGTCGCAAGGCTGAAACTT	TTGCCTCCAGCTTCCCTACA
Diplomonadida	277–285	15 (83.3%)	100% (1)	93.8% (1.1)	100.0%	RGGGACRGGTGAAATAGGATG	CAAATTGAGCCGCAGACTCC
Kinetoplastida	250–300	188 (95.9%)	92.2% (1.1)	83.2% (1.5)	96.7%	AAATTAAACCGCACGCTCCA	GCAAACGATGACACCCATGA
Microsporidia	370–440	96 (60.4%)	96.5% (1)	92.5% (1.2)	100.0%	BCAGGTTGATTCTGCCTGACR	ACCAGWCTTGCCCTCCARTT
Parabasalia	326–364	105 (85.4%)	100% (1)	96.8% (1)	100.0%	TAGGCTATCACGGGTAACGG	GCGTCCTGATTTGTTCACAG
Platyhelminthes	350–550	778 (56.2%)	96.6% (1.1)	88.7% (1.3)	30.3%	CAATTGGAGGGCAAGTCTGG	TGCTTTCGCWKTAGTTTGTCTG
Nematode A	320–335	1233 (70%)	87.7% (1.3)	68.1% (2)	99.2%	CACCCGTGAGGATTGACAG	CGATCACGGAGGATTTTCAA
Nematode B	380–410		96.4% (1.1)	82.2% (1.3)	99.9%	CGTCATTGCTGCGGTTAAAA	CCGTCCTTCGAACCTCTGAC
Nematode C	380–440		92.2% (1.1)	74.4% (1.7)	62.5%	AGTGGAGCATGCGGCTTAAT	TGCAATTCCCTRTCCCAGTC

The table shows, for each primer pair, expected amplicon lengths and a summary of the in silico assessment of the primer amplification range, information content, and specificity. The table indicates the number of species amplified in silico (and the proportion of species deposited in NCBI that this represents), the proportion of DNA sequences that matched a single genus/species (and the mean number of genera/species matching each DNA sequence), and the proportion of amplified DNA sequences belonging to the targeted taxon. The last two columns show the primer sequences

We evaluated the specificity and range of amplification of the selected primers using in silico PCR against all DNA sequences deposited in NCBI (see “Materials and Methods” for details). Overall, these primers captured between 56.3 and 95.4% of species within each targeted taxon (Table 1), though these estimates are likely conservative as the actual PCR conditions are less stringent than those in silico. Note also that these primers were designed to amplify putative pathogens: for example, the nematode primers only amplify, in silico, ~ 70% of all sequenced nematodes but should successfully amplify all common human pathogenic nematodes [38]. The ability to identify the organism carrying each DNA sequence differs among primer pairs and depends on the information content of the targeted DNA sequence, the number of annotated sequences deposited in the NCBI and the nomenclature of specific taxa. Most of the primers provide sufficient information to reliably identify at least the genus of most parasites amplified (Table 1). One of the Apicomplexan primer pairs that primarily amplifies *Cryptosporidium* spp. also shows low expected resolution at the species level, though this probably reflects the uncertain species boundaries within this genus. We also use the same evaluation pipeline to examine published generic eukaryote and parasite-specific primers [32, 33], though most of the resulting PCR products would too long to be sequenced on a high-throughput sequencer or did not display the desired sensitivity and specificity (Additional file 1: Table S2).

### Experimental assessment of the primers’ efficiency and specificity

We tested the sensitivity and specificity of the selected primers using genomic DNA directly extracted from parasites from the main taxa targeted (see the “Materials and Methods” section). After quality control, our dataset consisted of a total of 569,408 reads. All spiked-in parasites were correctly amplified and identified, even when a large amount of “contaminant” DNA (bacteria, mosquito, and human DNA) was mixed with the parasites’ DNA (Additional file 1: Table S3). Most primers exhibited strong preferential amplification of targeted parasite DNA. However, consistent with in silico analyses, the Amoebozoa, Platyhelminthes, and Nematoda C primers had larger proportions of off-target amplification (Table 1 and Additional file 1: Tables S3 and S4). For the other primers, we only observed a very small number of reads amplified from off-target species (typically around 5% of the reads with a taxonomy ID), despite the high ratio of contaminant/parasite DNA. The sequences mostly matched bacteria, but also included human, insect, and fungus sequences (Additional file 1: Table S4).

### Application to environmental and biological samples

**Fig. 1:**
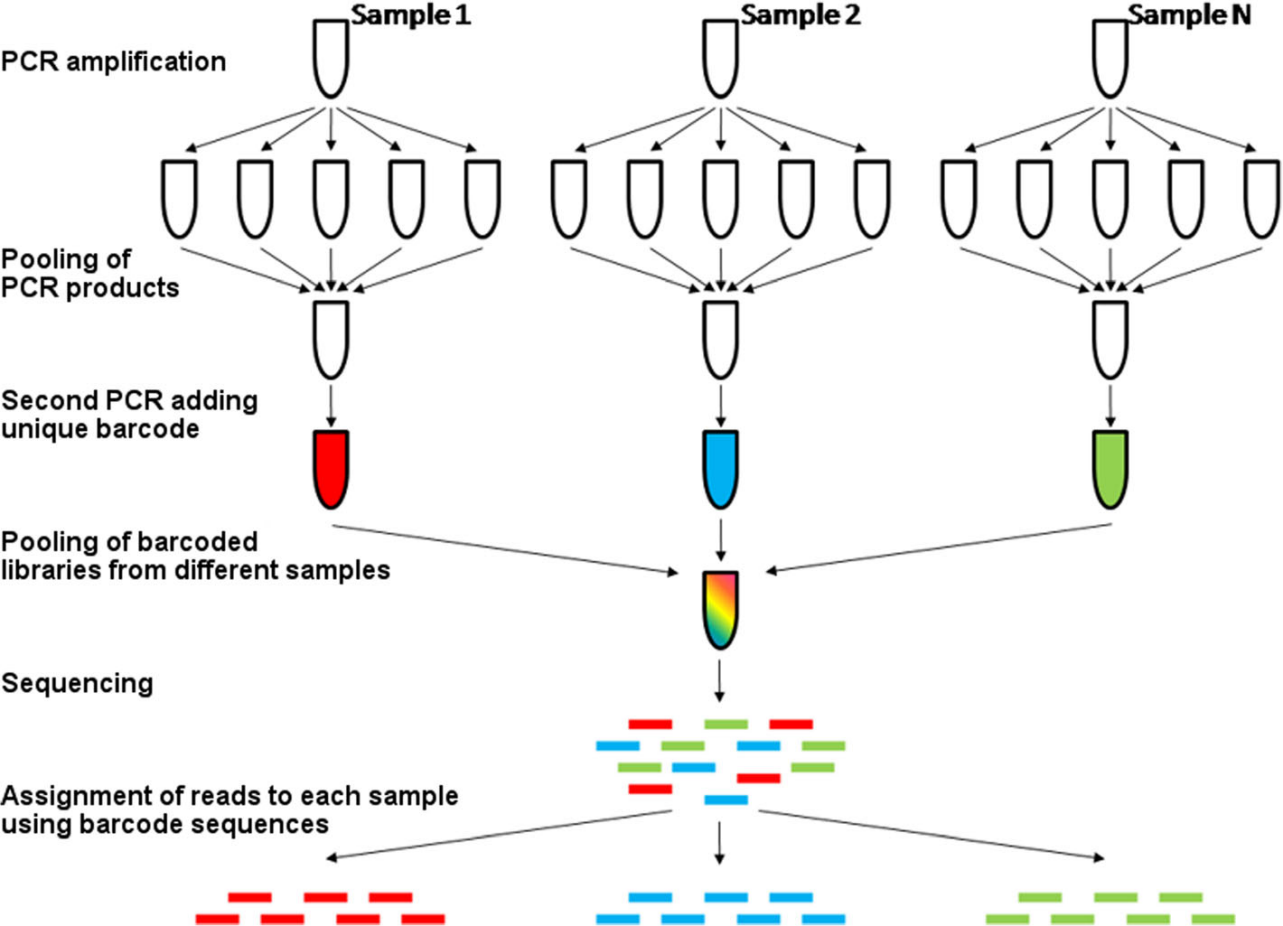
Overview of the assay. Schematic representation of the amplification, barcoding, and sequencing pipeline

We then tested this assay on DNA extracted from complex biological (human stools from individuals from Bangladesh, content of CO_2_-baited CDC light traps) and environmental samples (river water and soil samples). After amplification and sequencing (Fig. 1), we obtained DNA sequences from unicellular eukaryotes or helminthes from these samples for 11 of the 13 primer pairs tested (Table 2). Out of 83 water (i.e., no template) controls, only six yielded more than ten reads matching a unicellular eukaryote or helminth species, and with an average of 87 reads compared to an average of 1258 reads per parasite for the actual samples. These few reads could represent low-level laboratory contamination or reads incorrectly assigned to a sample due to sequencing errors in the barcode sequences [39].

**Table 2:** Examples of unicellular eukaryotes and helminthes identified in biological and environmental samples

Primer	Known parasite pool	Stool pools	Soil samples	Water samples	Trap contents
Amoebozoa	*Dictyostelium discoideum (100)*	*Endolimax nana (87)*	*Sorosphaerula veronicae (100)*	*Lecythium hyalinum (100)*	
		*Entamoeba hartmanni (100)*	*Filamoeba nolandi (97)*	*Phalansterium* sp. *(84)*	
		*Entamoeba dispar (100)*	*Dictyamoeba vorax (89)*	*Stramenopile* sp. *(99)*	
			*Polymyxa graminis (99)*	*Kraken carinae (98)*	
			*Cercozoa* sp. *(97)*	*Leptophryidae* sp. *(97)*	
Apicomplexa B	*Cryptosporidium* sp. *(100)*				*Plasmodium gallinaceum (98)*
					*Plasmodium cathemerium (99)*
					*Plasmodium juxtanucleare (94)*
Apicomplexa C	*Theileria parva (100)*		*Paraschneideria metamorphosa (97)*		
	*Cryptosporidium* sp. *(100)*				
*Blastocystis*		*Blastocystis* sp. *(100)*			
Diplomonadida	*Giardia intestinalis (100)*	*Enteromonas hominis (100)*		*Hexamita inflata (97)*	
		*Giardia intestinalis (100)*		*Hexamita nelsoni (94)*	
				*Trepomonas steinii (96)*	
				*Trepomonas* sp. *(94)*	
				*Hexamita inflata (99)*	
Kinetoplastida	*Leishmania* sp. *(100)*		*Rhynchomonas nasuta (94)*	*Rhynchomonas nasuta (100)*	*Crithidia dedva (93)*
	*Trypanosoma brucei (100)*		*Bodonidae* sp.*/Neobodo designis (79)*	*Neobodo designis (90)*	*Crithidia* sp.*/Leptomonas* sp.*/Wallaceina* sp. *(99)*
			*Cryptaulax* sp. *(95)*	*Procryptobia sorokini (99)*	*Herpetomonas* sp.*/Herpetomonas isaaci (89)*
			*Phanerobia pelophila/Dimastigella trypaniformis (93)*	*Parabodo caudatus (99)*	
			*Dimastigella trypaniformis (98)*	*Bodo saltans (100)*	
Microsporidia	*Encephalitozoon cuniculi (100)*	*Enterocytozoon bieneusi (100)*		*Microsporidium* sp. *(95)*	*Pleistophora* sp. *(100)*
					*Microsporidium* sp. *(98)*
Nematoda A	*Acanthocheilonema viteae (100)*	*Enterobius vermicularis (100)*	*Mesocriconema* sp. *(100)*		*Abursanema iranicum (95)*
			*Filenchus* sp.*/Tylenchidae* sp. *(100)*		
Nematoda B	*Acanthocheilonema viteae (100)*				*Abursanema iranicum/Sphaerularia vespae (92)*
Nematoda C			*Diphtherophora* sp. *(99)*		*Mermithidae* sp. *(92)*
			*Prismatolaimus* sp. *(100)*		
			*Alaimus parvus (99)*		
			*Pellioditis* sp. *(99)*		
			*Oscheius* sp. *(100)*		
Platyhelminthes	*Schistosoma* sp. *(99)*			*Cura pinguis (98)*	
				*Girardia tigrina (92)*	

The table shows, for each type of sample and each primer pair, up to five species best matching DNA sequences amplified (and the percentage identify with the most similar NCBI sequence). When one DNA sequence matched equally well multiple genera, all those are indicated. The full results are presented in Additional file 1: Table S4

**Fig. 2:**
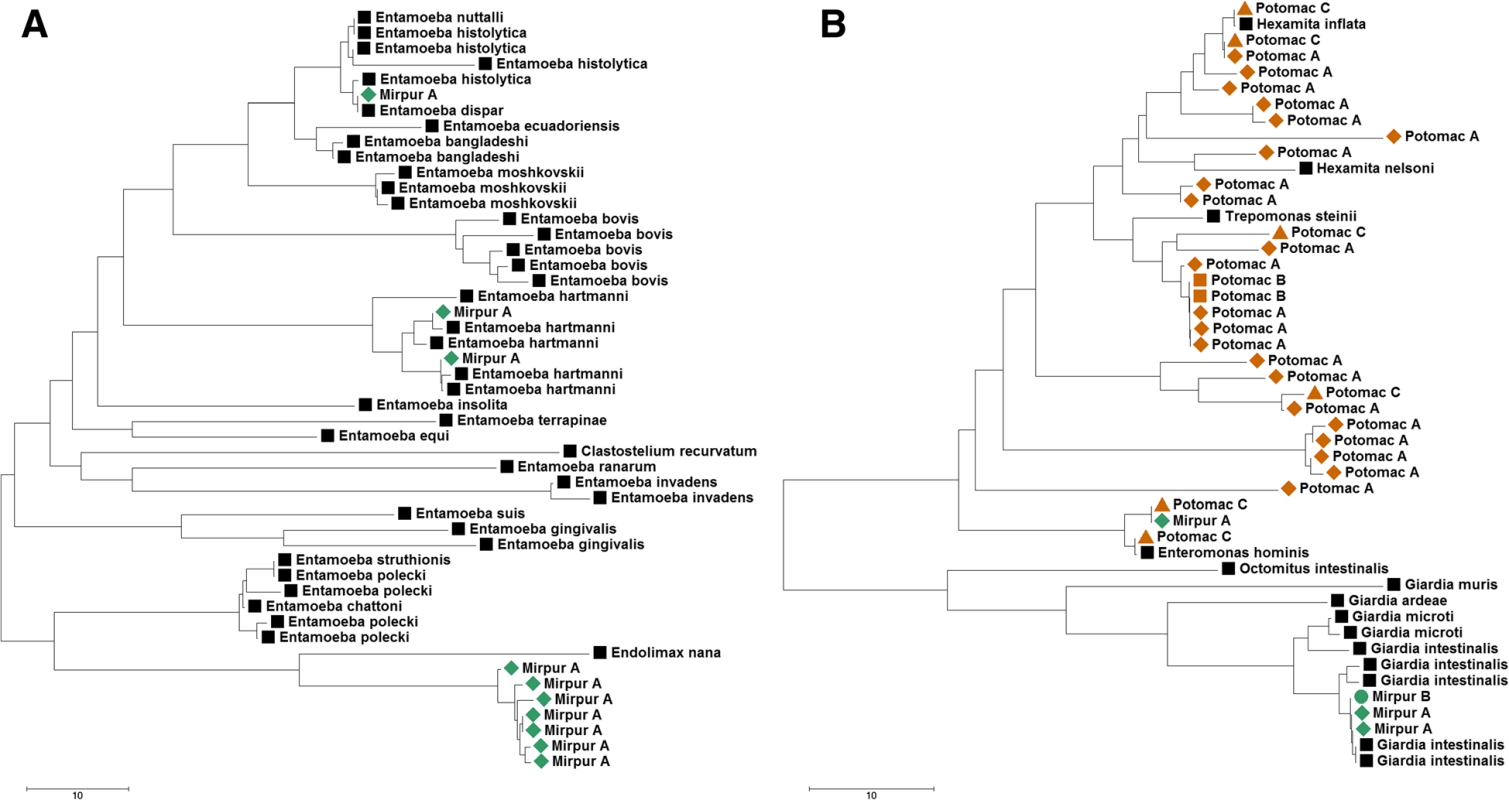
Phylogenetic reconstruction showing the relationship of amplified sequences with annotated NCBI sequences. **a** Neighbor-joining tree showing the relationships among annotated Amoebozoa sequences (black squares) and those amplified from human stool samples (green diamonds). **b** Neighbor-joining tree showing the relationships among, and diversity of, annotated Diplomonadida sequences (black squares) and those amplified from pooled stool (green diamonds and circles) and three Potomac River samples (brown shapes)

From the pools of DNA extracted from ~ 300 human stool samples from Bangladesh, the Amoebozoa primers yielded three distinct DNA sequences. One sequence was identical to *Entamoeba hartmanni* and another identical to *Entamoeba dispar*, two common commensal of the human gut [40]. The third sequence amplified was most similar to *Endolimax nana*, but with only 87% identity (Fig. 2a) and likely originated from a related Amoebozoa that has not yet been sequenced at this locus (possibly an *Iodamoeba* species). We also identified DNA sequences identical to *Blastocystis*, a common parasite of the human gut with unclear clinical consequences [41, 42]. The Diplomonadida primers yielded DNA sequences identical to *Enteromonas hominis*, a likely non-pathogenic flagellate, as well as sequences of *Giardia intestinalis*, a water- and food-borne pathogen that can cause severe diarrhea [43]. Finally, we identified DNA sequences from *Enterocytozoon bieneusi*, a microsporidian parasite causing diarrhea [44], and from *Enterobius vermicularis*, a common pinworm (Table 2).

In the water and soil samples, we amplified DNA sequences from many free-living unicellular eukaryotes (e.g., *Lecythium hyalinum*, *Rhynchomonas* sp., *Bodo saltans*) and helminths (e.g., *Cura* sp., *Prismatolaimus* sp.) (Table 2). In addition, we identified DNA sequences most similar to obligate plant parasites, including *Polymyxa graminis*, a parasite responsible for the transmission of important crop viruses [45]. Finally, we identified, in the water samples, sequences most similar to those of organisms typically found in animal gut (e.g., *Enteromonas hominis*) and numerous uncharacterized species within the order Diplomonadida that include free-living and parasitic organisms (Fig. 2b), illustrating how this approach could be used for monitoring water quality or safety.

Finally, the DNA extracted from the entire content of CO_2_-baited light traps yielded a large number of DNA sequences from known parasites of insects (e.g., *Crithidia* sp., *Mermithidae* sp.) as well as from bird parasites transmitted by blood-sucking insects (e.g., *Plasmodium gallinaceum*) (Table 2).

## Discussion

We described here a novel sequencing-based assay, akin to the bacterial 16S rRNA sequencing [31], that provides a high-throughput and comprehensive platform for detecting and identifying many eukaryotic parasites and closely related non-parasitic organisms, including most Apicomplexans, Amoebozoa, Kinetoplastids, Nematodes, and Platyhelminthes. This assay could efficiently complement current clinical or research assays that typically target a single pathogen at a time or rely on low-throughput and low-resolution microscopic analyses. Similar approaches have been proposed previously using generic Eukaryote primers, but these often suffers from important limitations. First, these eukaryote primers are likely to also amplify overwhelming “contaminating” DNA, such as human or mosquito DNA that could swamp the signal from microorganisms. Second, due to a lack of adequate computational tools to evaluate primers, it is not clear that these generic primers actually amplify the taxa of interest (see, e.g., Additional file 1: Table S2), especially in the presence of many different eukaryotic DNA sequences. By contrast, our assay relies on primers designed to amplify specific taxa with little off-target, enabling increased sensitivity and high level of multiplexing resulting in a low cost per sample and a high throughput (see below).

One key feature of our assay compared to current detection methods, is that it enables distinguishing closely related species that often have very different clinical implications: for example, the *Entamoeba* sequences identified in the stool samples were unambiguously assigned to *E. hartmanni* and *E. dispar*, two non-pathogenic organisms related to, but distinct from, the pathogenic *E. histolytica* (Fig. 2a). Further, because this detection method is not targeting specific species but selected taxonomic groups, it enables detection of previously uncharacterized organisms (e.g., Fig. 2b). The assay is currently implemented in 384-well plate format and supports the simultaneous analysis of more than 350 samples (plus controls) for less than US$5500 total (or ~ US$15 per sample). Note that the number of samples pooled on one sequencing run can likely be increased for most projects, further decreasing the cost per sample, though this will depend on the samples’ expected diversity. For example, analysis of 384 samples for 10 amplicons can be performed on one run of an Illumina MiSeq and generate, on average, more than 5000 reads per amplicon per sample, enabling detection of even low abundance parasites. The high throughput and low cost per sample are the key advantages of this approach compared to metagenomic approaches [46] in which the entire DNA pool is sequenced without any selection: metagenomics approaches typically suffer from the sequencing of overwhelming host and/or bacterial DNA which (1) reduces the parasite signal and (2) dramatically increases the amount of sequencing required from each sample (and therefore the cost) to detect even fairly abundant parasites.

We believe the sequencing assay described here could be extremely useful to study unicellular eukaryotes and helminths in a wide variety of settings. For example, by allowing rapid screening of large numbers of human biological samples, it could, simultaneously, support rigorous and well-powered analyses of the clinical consequences of commonly reported parasites, and identification of rare but clinically important parasites. This assay can also complement existing microbiome studies to provide a comprehensive perspective on the microorganisms present in an environment and provide a foundation to better understand their interactions. Another exciting application of this assay would be vector-borne disease surveillance. Many eukaryotic parasites transmitted by mosquitoes, flies or ticks cause significant morbidity and mortality in endemic areas. Unfortunately, entomological surveillance strategies are resource-intensive and, therefore, typically limited to the most urgent threats. This assay could provide a significant improvement by allowing simultaneous screening of very large numbers of vectors (e.g., the content of more than 350 insect traps at once) for different types of pathogens, including emerging or uncharacterized threats. Finally, the assay could be easily deployed to monitor animal health (e.g., livestock, bees, fish farming) and environmental or food safety.

One final important feature of the assay described here is its customizability. Since it relies on PCR amplification, it is easy to modify it to include additional taxa (e.g., viruses). In this regard, it is important to note that all primers described here are located within genic regions and can therefore be used to amplify cDNA synthesized from RNA (see also below).

One cautionary note is that this assay, similarly to bacterial 16S rRNA sequencing, could fail to detect rare parasites present in the original sample but stochastically lost in the subsample used in the PCR or below the sensitivity limit of the PCR amplification. This issue is partially alleviated by amplification of a gene (18S rRNA) that is present in multiple copies in each parasite cell and could be further reduced by using reverse transcriptase PCR, which would dramatically improve sensitivity since a very large number of rRNA copies are present in each cell. Note however that, while the laboratory protocols required to process RNA instead of DNA are straight-forward, RNA-based analyses require sample collection and storage protocols that may not possible for all studies.

## Conclusions

Overall, the assay described here provides a novel method to comprehensively characterize parasites and many other unicellular eukaryotes and helminthes, from a wide variety of samples and could complement existing bacterial studies to significantly improve our understanding of the role of the microbiome in studies of human and animal health.

## Methods

### PCR primer design

We designed PCR primers to amplify 18S rRNA genes from most eukaryotic taxa containing common human parasites [37]: Apicomplexa, Amoebozoa, *Blastocystis*, Diplomonadida, Nematoda, Platyhelminthes, Kinetoplastida, Parabasalia, and Microsporidia (Additional file 1: Table S1). For our purpose, the primers needed to fulfill several criteria: (i) they had to amplify all species within a taxon of interest while having little off-target amplification (especially avoiding amplification of mammalian or arthropod DNA), (ii) they had to provide enough genetic information to reliably identify the organism carrying each DNA sequence, and (iii) the amplified products had to be short enough to be sequenced using Illumina chemistry. To generate primers satisfying these specific constraints, we first downloaded all DNA sequences for the targeted gene within the selected taxon from the NCBI nucleotide database. We randomly kept a single DNA sequence per species and discarded all sequences generated from organisms not fully annotated at the species level (including all environmental samples). We then retrieved the full gene annotation for each sequence and used this information to trim longer sequences to only the targeted gene. Next, we aligned all sequences using MAFFT [47] and generated a consensus DNA sequence, using ambiguity codes for positions variable in at least 20% of the sequences. We used this consensus sequence as input for primer3 [48] and generated primers allowing for up to two ambiguous bases per primer, an annealing temperature between 57 and 63 °C, and an amplicon length between 200 and 450 bp. Since some taxa were highly diverse, we had to design multiple, complementary primer pairs to efficiently capture most species, leading to a total of 13 primer pairs to amplify nine taxa (Table 1).

### In silico evaluation of primer pairs

We performed extensive in silico evaluation of each primer pair to assess their specificity, amplification range and information content (Additional file 1: Figure S2 and https://github.com/MVesuviusC/2018_methods_paper). We also assessed selected primers from the literature using the same pipeline (Additional file 1: Table S2).

To determine what organisms could be amplified with each primer pair, we ran PrimerTree [49] restricting the search to the targeted taxon and retrieving up to 10,000 DNA sequences with the corresponding primer sites from NCBI. The PrimerTree results were used to generate representative phylogenetic trees (Additional file 1: Figure S3), to estimate the range of the amplicon lengths, and to determine the numbers of genera and species for which DNA sequences could be amplified with a given primer pair (excluding all annotations containing “sp.,” “uncultured,” “unidentified,” “cf.,” “isolate,” or “symbiont”).

To determine the information content of each amplicon, we exported all DNA sequences retrieved from PrimerTree (excluding incompletely annotated sequences as described above), trimmed the primer sequences and kept a single occurrence of each species/sequence combination (keeping multiple sequences per species if they differed). We further discarded any sequence shorter than 150 bp or longer than 500 bp as these would be lost during library preparation or analysis (the maximum length of 500 bp was not used to evaluate primers from the literature). We then compared each remaining DNA sequence to all sequences in the NCBI nt database using BLAST, allowing up to 10,000 matches and determined the number of genera and species with sequences identical to each queried sequence.

To identify species of the targeted taxon that would be missed due to nucleotide differences in the primer sites, we compared the list of species obtained by PrimerTree (i.e., using in silico PCR) with the list of species identified by blasting the entire amplicon sequences as described above (and making sure these sequences were long enough to include sequences on the 5′ and 3′ ends at least as long as the primers).

Finally, we assessed the specificity of the primers by re-running PrimerTree without any taxonomic restriction and calculated the proportion of sequences retrieved belonging to the targeted taxon.

### Samples analyzed

To experimentally test the primer pairs, we obtained genomic DNA from the Biodefense and Emerging Infections (BEI) Research Resources Repository for the following species: *Trypanosoma brucei* (NR-49828), *Giardia intestinalis* (NR-15894), *Cryptosporidium parvum* (NR2519), *Leishmania tropica* (NR-50127), *Encephalitozoon cuniculi* (NR-13510), *Schistosoma mansoni* (NR28910), and *Acanthocheilonema viteae* (NR-48884). Genomic DNA from *Dictyostelium discoideum* and *Theileria parva* were kindly provided by Drs. O’Connor and Carneiro Da Silva. All parasite DNAs were mixed in roughly equal concentration to generate a single pool that was then either analyzed independently or mixed together with DNA from *E. coli*, human, and *Anopheles* DNA. Since some samples contained host DNA, the exact concentration of each parasite DNA is unknown.

We also analyzed uncharacterized biological and environmental samples. We extracted DNA from four soil samples collected near Baltimore, MD (approximatively 0.25 g each), which we diluted both 1:10 and 1:100 to avoid PCR inhibition. We extracted three water samples collected in the Potomac river (approximately 50 mL each) using the PowerSoil DNA isolation kit (Qiagen). We also extracted DNA from the entire content of three CDC CO_2_-baited light traps placed overnight in suburban areas of Maryland. Lastly, we pooled human stool DNA samples collected in Mirpur, Bangladesh, into two pools (~ 96 each).

### PCR amplification and high-throughput sequencing

We amplified DNA extracted from each sample (as well as 83 negative controls) with each primer pair using the GoTaq® DNA polymerase (Promega) under the following conditions: initial denaturing step at 95 °C followed by 40 cycles of 95 °C for 30 s, 50 °C for 30 s, and 72 °C for 30 s. A final extension at 72 °C for 10 min was followed by incubation at 4 °C. For the *Blastocystis*, Apicomplexa A, Parabasalia, and Nematoda B primers that generated large amount of primer dimers when no DNA template was present, we spiked each PCR reaction with 0.01 ng of an artificial construct to decrease primer dimerization and digested it before sequencing (see “Prevention of dimer formation using artificial construct template” below). We then pooled all PCR products generated from one DNA sample and performed a second PCR to incorporate at the end of each molecule (i) a unique oligonucleotide “barcode” specific to each sample and (ii) DNA sequences complementary to the Illumina sequencing primers (Fig. 1) [49, 50]. We then pooled all resulting barcoded libraries (each containing all PCR products amplified from each sample) and sequenced them simultaneously on an Illumina HiSeq 2500 to generate 300 bp paired-end reads.

### Bioinformatic analyses

We first separated the reads generated from each sample according to their unique oligonucleotide barcodes (Fig. 1). We trimmed 50 low-quality bases from the 5′ end of each read. We also discarded shorter-than-expected sequences (e.g., primer dimers) by identifying reads for which the last 20 bp had an average read quality below 20. We then merged overlapping ends of each read pair using PANDAseq [51] to generate, from each read pair, a single consensus DNA sequence and correct sequencing errors that disproportionally occur at the end of the reads. All read pairs that did not merge correctly were discarded from further analyses. We identified and trimmed the primer sequences from each read and eliminated all reads shorter than 150 bases as they likely represent experimental artifacts (e.g., PCR chimeras and primer dimers). After combining reads from all samples, we kept a single copy of each unique DNA sequence and recorded which reads from each sample carried each of these unique DNA sequences. Sequences observed less than 20 times in the entire dataset were discarded as they likely resulted from sequencing errors [49]. We then compared each unique DNA sequence to all sequences deposited in the NCBI nt database using BLAST and used custom code developed in our laboratory to retrieve the taxonomic information associated with the most similar sequence(s) (https://github.com/MVesuviusC/2018_methods_paper). Only sequences with more than 70% identity over the entire sequence length were further considered. If DNA sequences from multiple species were equally similar to one of our sequences, we recorded all corresponding species names. Finally, we summarized, for each sample, the parasite species identified, the percentage identity between the reads and the most similar NCBI sequence(s), and the number of reads supporting the identification in this sample.

### Phylogenetic analyses

To better characterize specific DNA sequences with ambiguous taxonomic identification, we analyzed them with orthologous sequences from closely related species. Briefly, we used PrimerTree [49] to retrieve orthologous DNA sequences from NCBI from species of the targeted taxon and aligned them with the ambiguously assigned DNA sequence(s) using MAFFT [47]. We then reconstructed neighbor-joining trees to determine the phylogenetic relationships of the amplified DNA sequences using MEGA [52].

## Additional file

Additional file 1: **Table S1.** Examples of genera targeted by each primer pair. **Table S2.** Primer characteristics of primers from the literature. **Table S3.** Amplification of positive controls. The table shows the results of the sequencing assay when performed on pools of DNA from known parasites, with and without addition of *Anopheles/Escherichia*/Human DNA. Each column shows the percentage of the reads that match each observed species. Green text represents on-target species and red text shows off-target (often host) species amplification. **Table S4.** Results from all samples. Explanation of each column is presented in the excel sheet. **Figure S1.** Complementarity of the primer pairs targeting the same taxonomic groups. *Apicomplexa* and *Nematoda* each required three primer pairs to capture the diversity within these groups. The taxa amplified by each of the three primer sets are presented as Venn diagrams showing the overlap in species coverage. The percent within each sector is shown in parentheses. **Figure S2.** Overview of the pipeline for the in silico assessment of the primer amplification range, information content and specificity. **Figure S3.** PrimerTree results for each newly designed primer. The figures show, for each primer set, the PrimerTree plot and amplicon lengths. The PrimerTree results were restricted only to the targeted group to show the diversity of on-target taxonomic groups amplifiable. (ZIP 2497 kb)

## Abbreviation

rRNA: Ribosomal RNA
