# Supplementary material for: A high-throughput sequencing assay to comprehensively detect and characterize unicellular eukaryotes and helminths from biological and environmental samples
Source: Microbiome. 2018 Oct 29;6:195. doi: 10.1186/s40168-018-0581-6 (PMC6206884; doi:10.1186/s40168-018-0581-6)
Supplement: Supplementary file 1 — Table S1. Examples of genera targeted by each primer pair. Table S2. Primer characteristics of primers from the literature. Table S3. Amplification of positive controls. The table shows the results of the sequencing assay when performed on pools of DNA from known parasites, with and without addition of Anopheles/Escherichia/Human DNA. Each column shows the percentage of the reads that match each observed species. Green text represents on-target species and red text shows off-target (often host) species amplification. Table S4. Results from all samples. Explanation of each column is presented in the excel sheet. Figure S1. Complementarity of the primer pairs targeting the same taxonomic groups. Apicomplexa and Nematoda each required three primer pairs to capture the diversity within these groups. The taxa amplified by each of the three primer sets are presented as Venn diagrams showing the overlap in species coverage. The percent within each sector is shown in parentheses. Figure S2. Overview of the pipeline for the in silico assessment of the primer amplification range, information content and specificity. Figure S3. PrimerTree results for each newly designed primer. The figures show, for each primer set, the PrimerTree plot and amplicon lengths. The PrimerTree results were restricted only to the targeted group to show the diversity of on-target taxonomic groups amplifiable. (ZIP 2497 kb) [file 40168_2018_581_MOESM1_ESM.zip › SFigure_3.pdf]

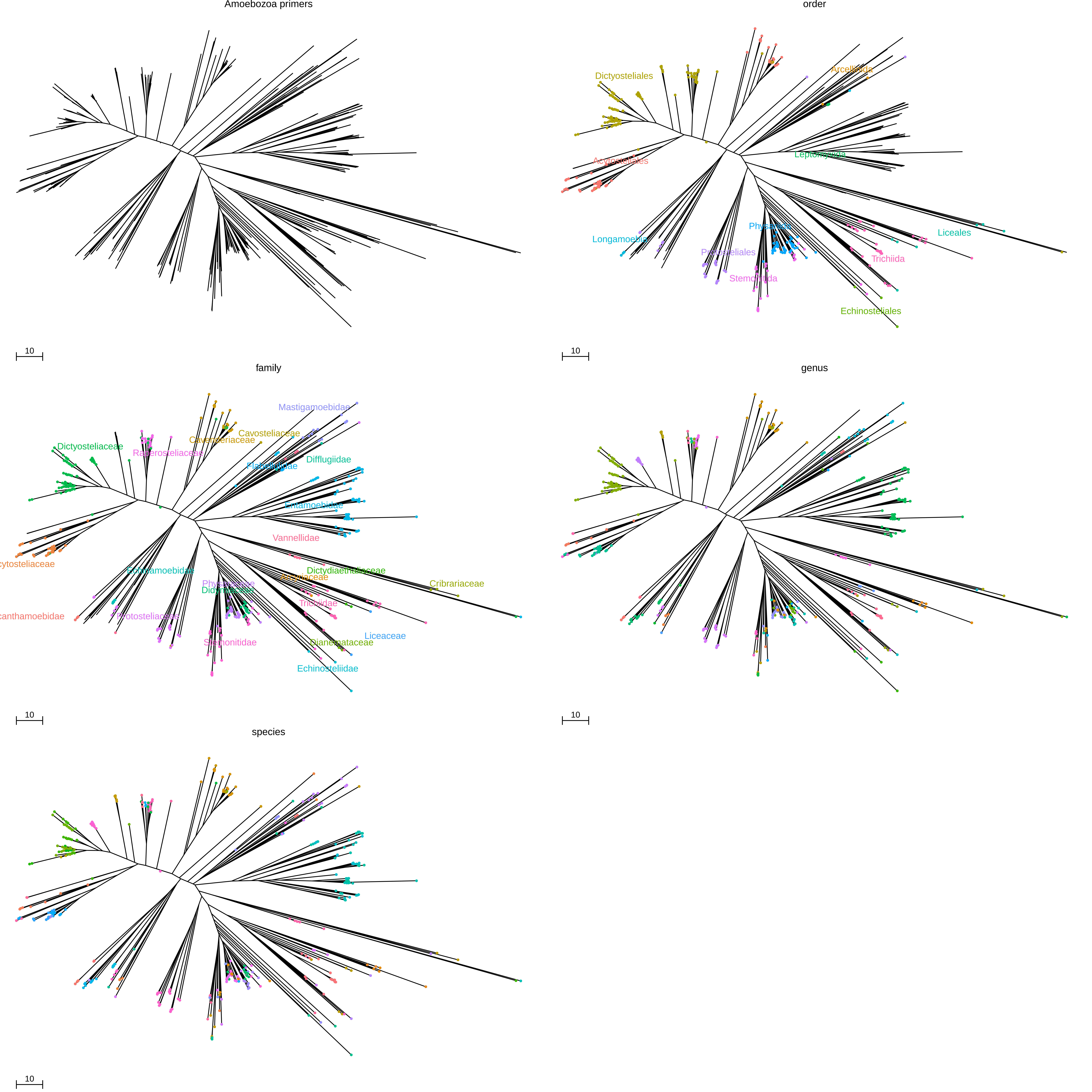
Amoebozoa primers
10
order
Dictyosteliales
Arcellinida
Acytosteliales
Leptomyxida
Longamoebia
Physarida
Protosteliales
Liceales
Trichiida
Stemonitida
Echinosteliales
10
family
Mastigamoebidae
Dictyosteliaceae
Cavosteliaceae
Cavenderiaceae
Raperosteliaceae
Difflugiidae
Flabellulidae
Entamoebidae
Vannellidae
Echinamoebidae
Dictydiaethaliaceae
Arcyriaceae
Physaraceae
Didymiaceae
Cribrariaceae
Protosteliaceae
Trichiidae
Stemonitidae
Liceaceae
Dianemataceae
Echinosteliidae
10
genus
10
species
10

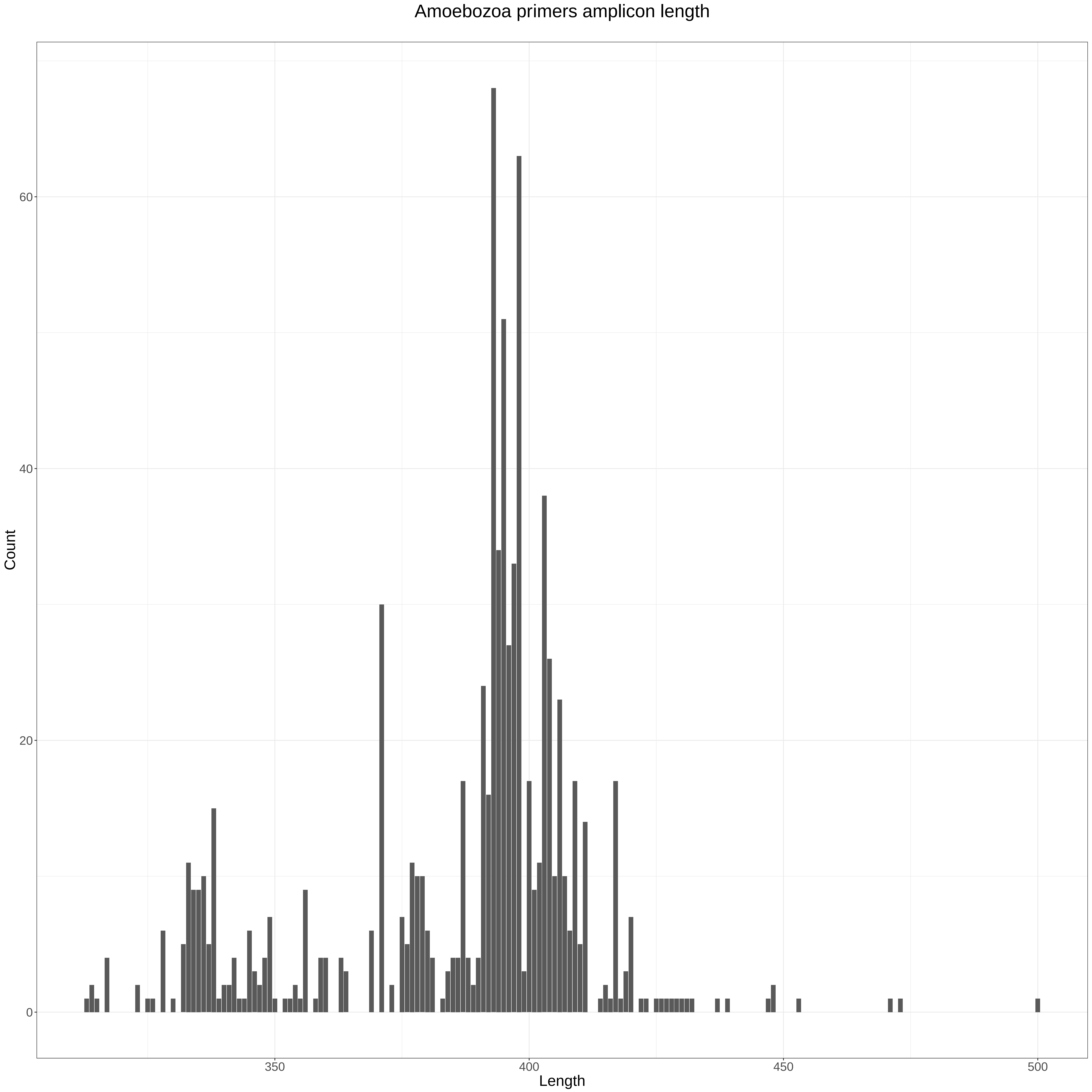

Amoebozoa primers amplicon length
Count
60
40
20
0
350
400
450
500
Length

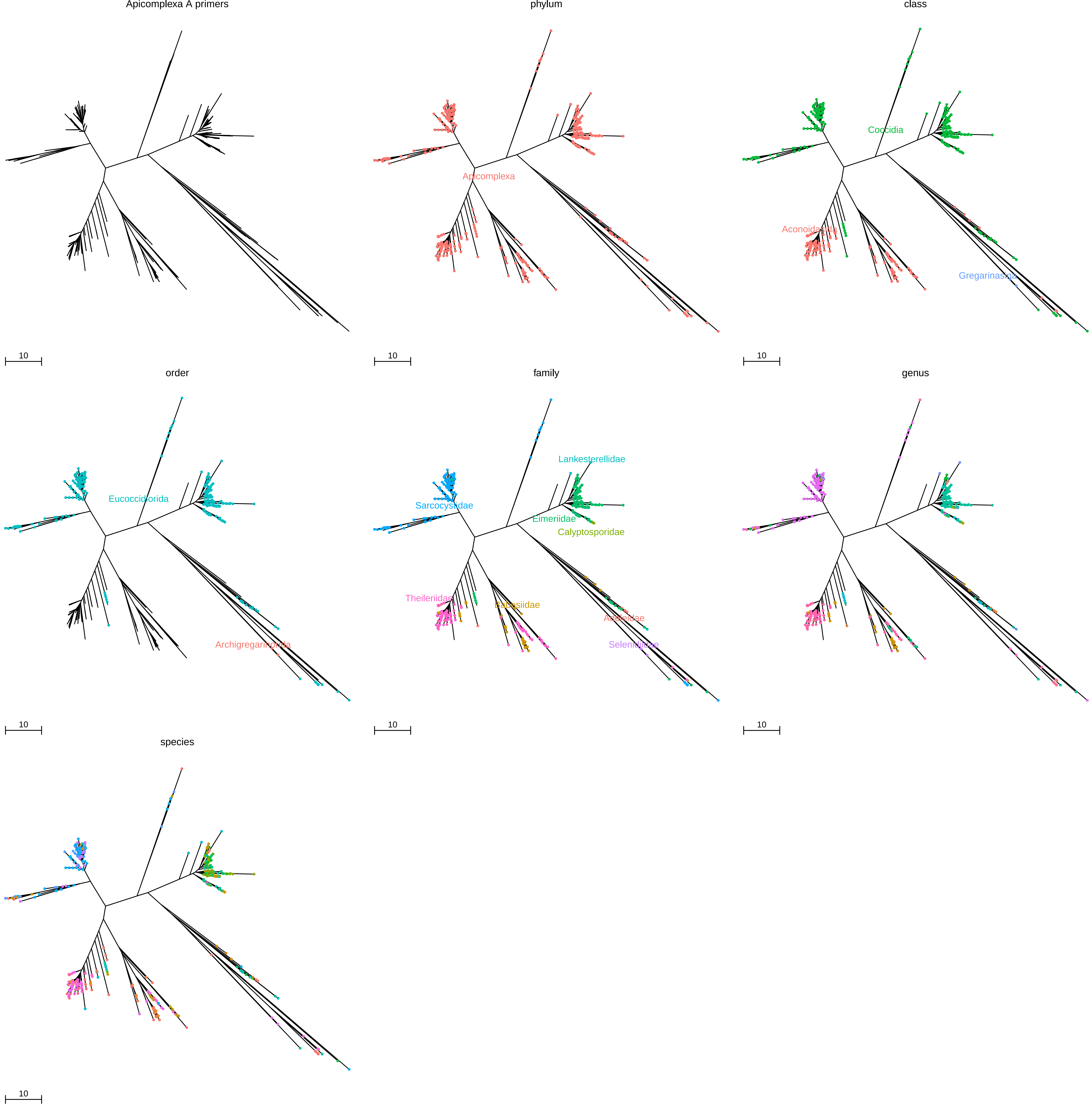

Apicomplexa A primers
phylum
class
Apicomplexa
Coccidia
Aconoidasida
Gregarinasina
order
family
genus
Eucoccidiorida
Archigregarinorida
Lankesterellidae
Sarcocystidae
Eimeriidae
Calyptosporidae
Theileriidae
Babesiidae
Adeleidae
Selenidiidae
species
10

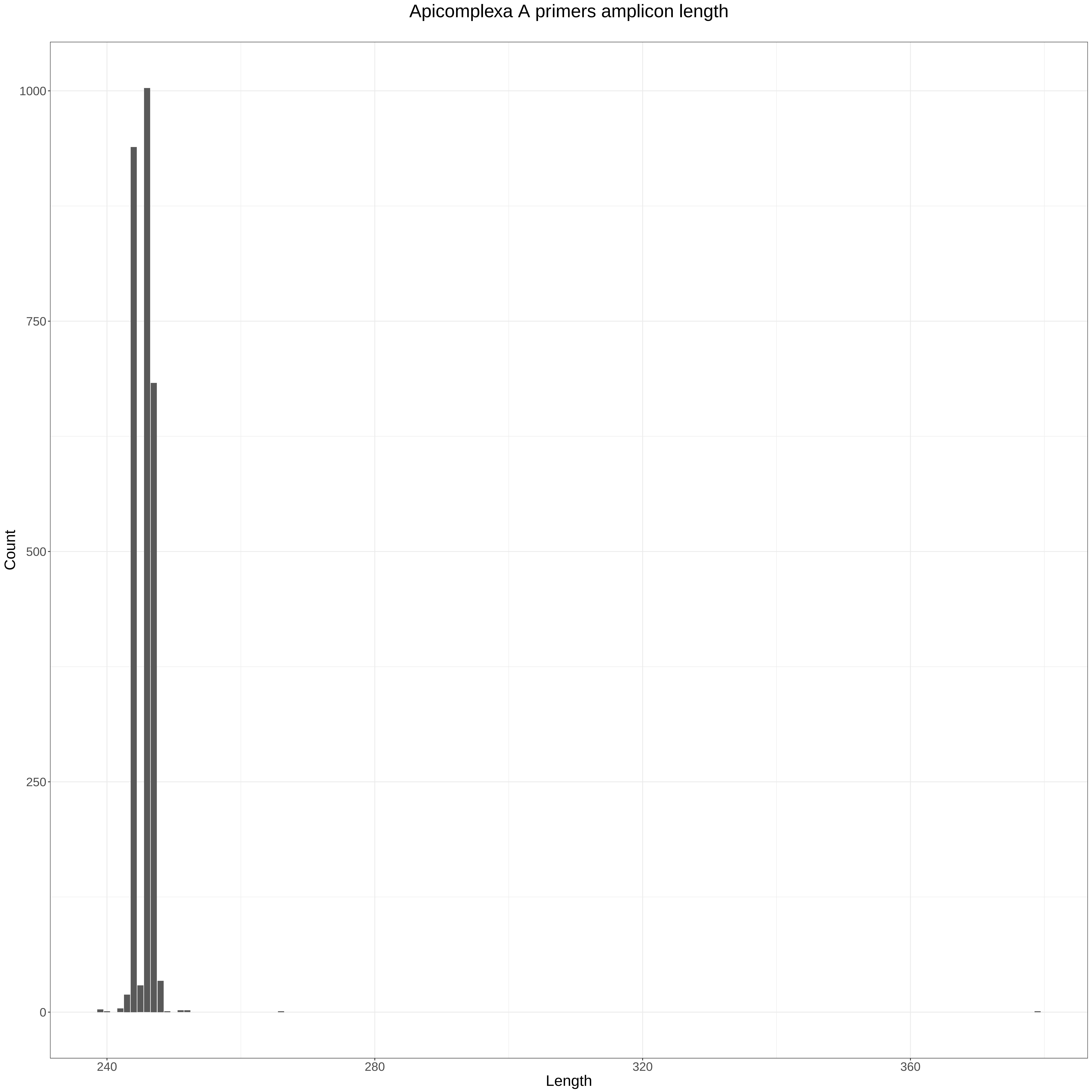
Apicomplexa A primers amplicon length
Count
1000
750
500
250
0
240
280
320
360
Length

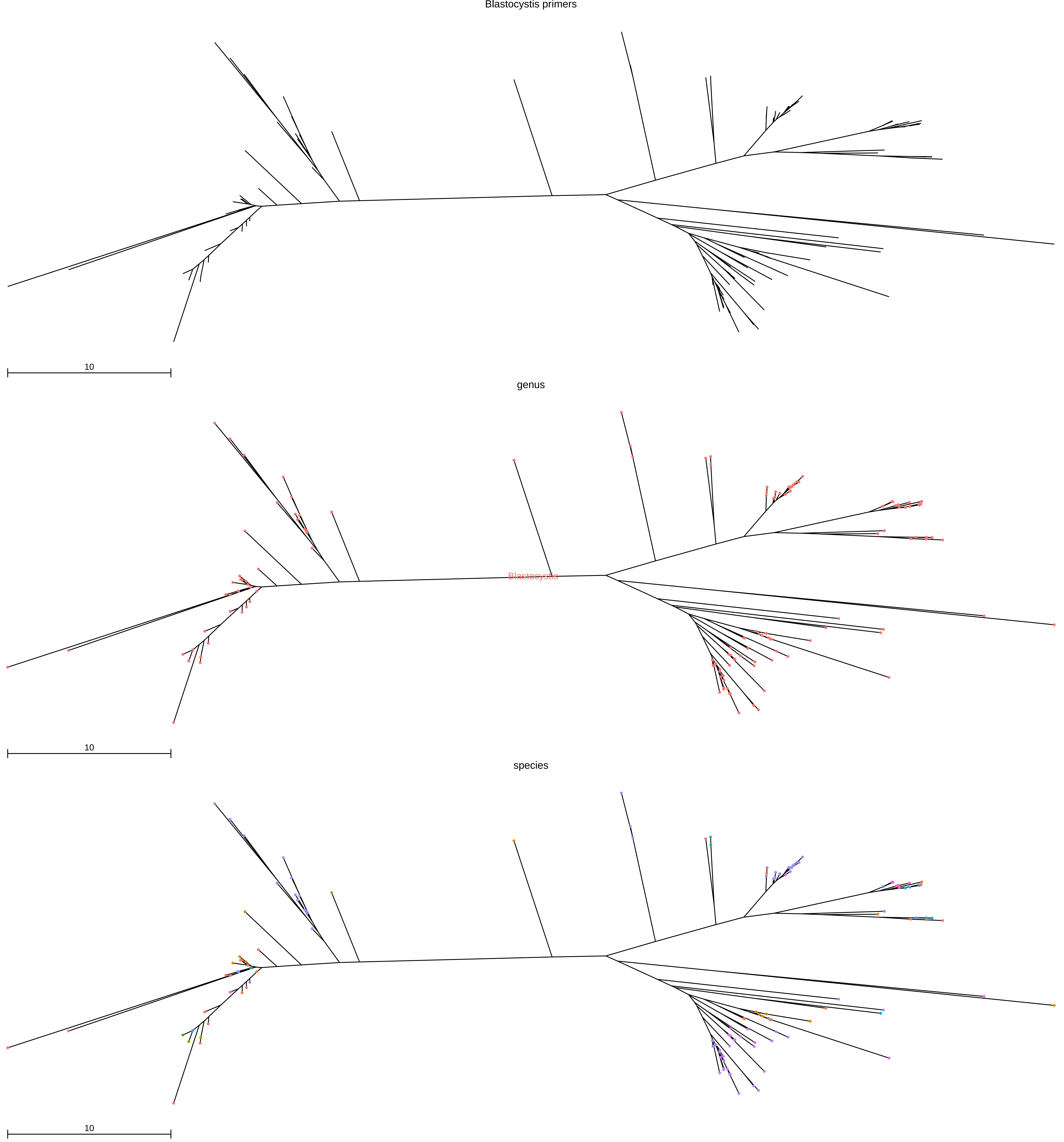
Blastocystis primers
10
genus
Blastocystis
10
species
10

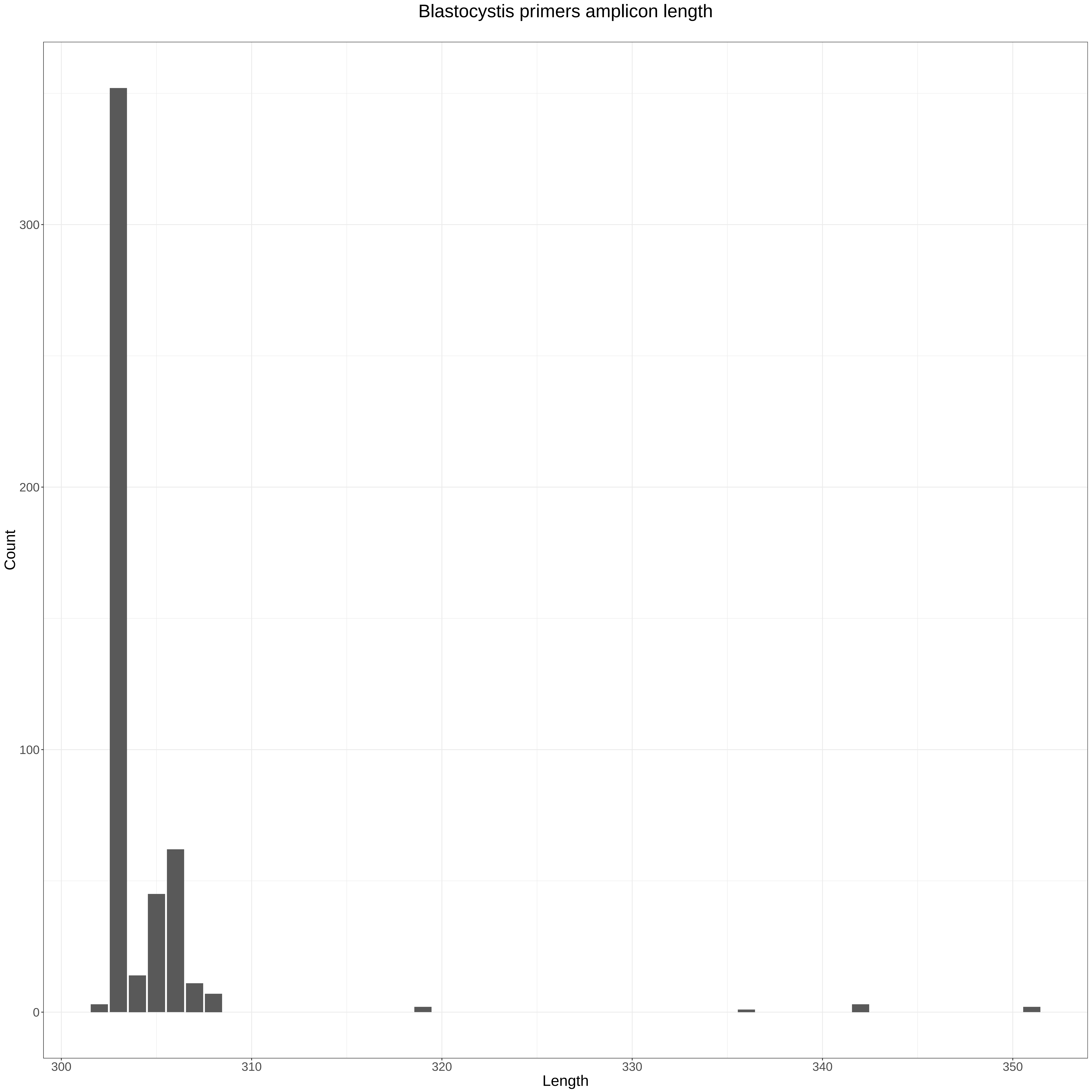
Blastocystis primers amplicon length
Count
Length
0
100
200
300
300
310
320
330
340
350

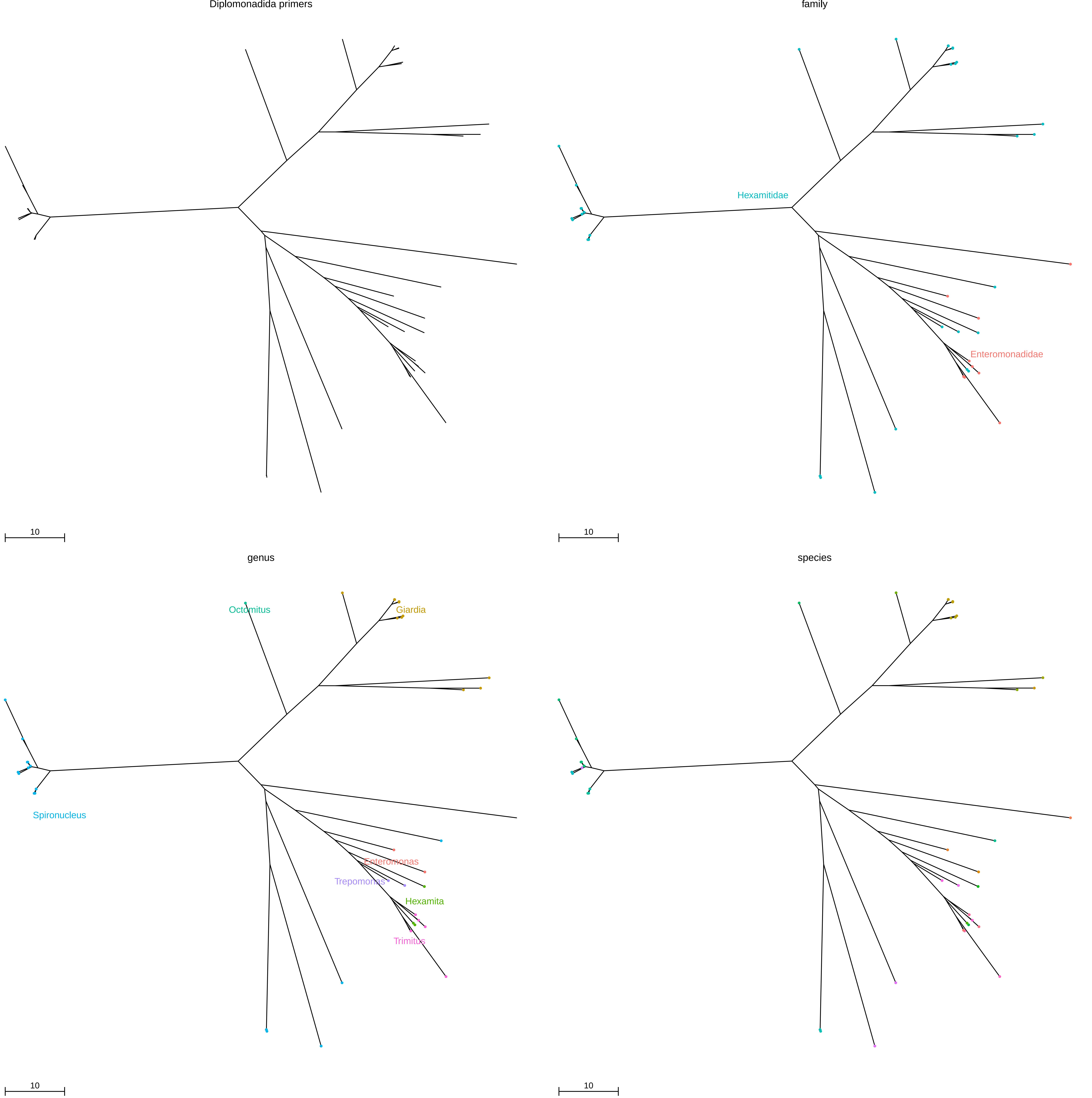

Diplomonadida primers
10
family
Hexamitidae
Enteromonadidae
10
genus
Octomitus
Giardia
Spironucleus
Enteromonas
Trepomonas
Hexamita
Trimitus
10
species
10

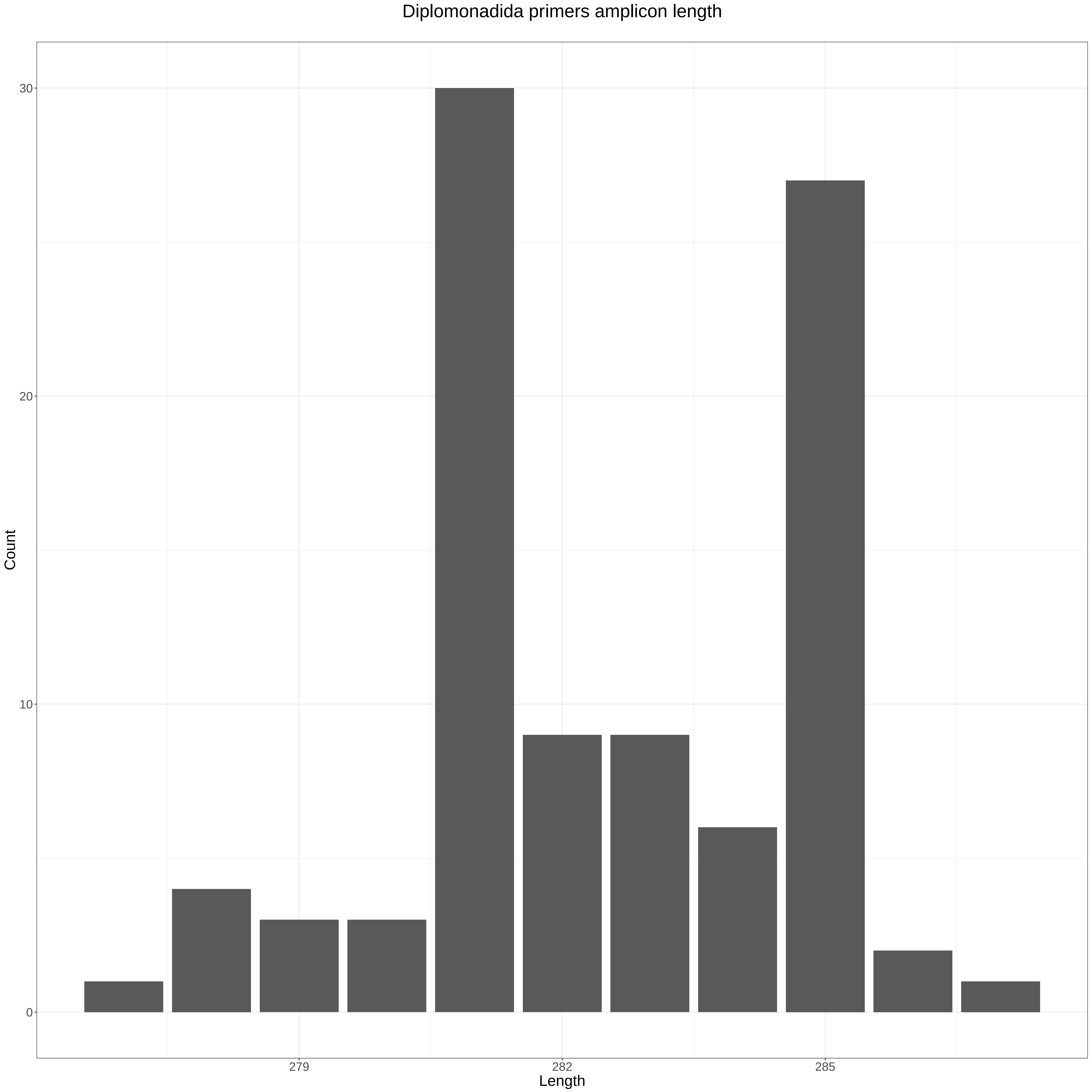
Diplomonadida primers amplicon length
Count
Length
0
10
20
30
279
282
285

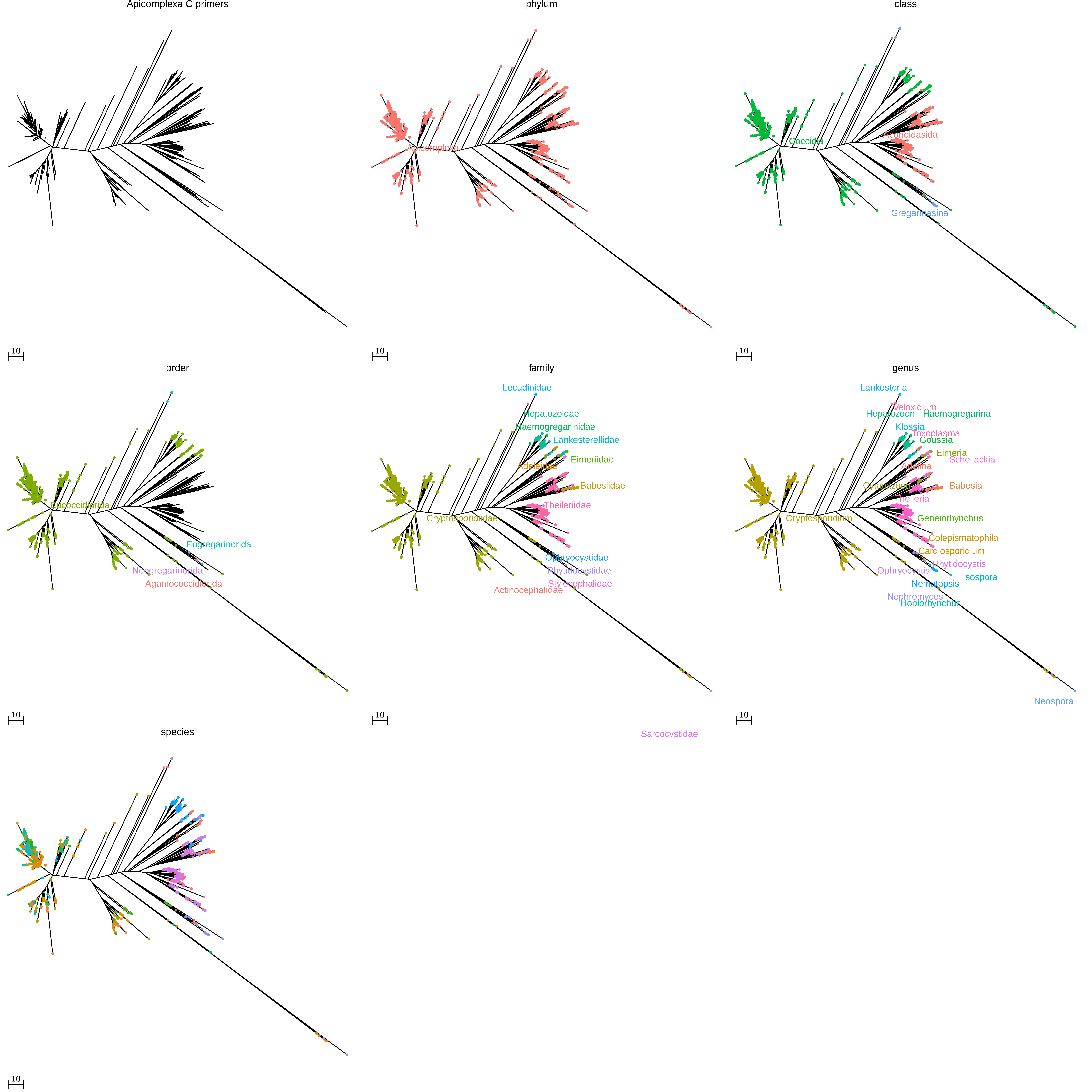

Apicomplexa C primers
phylum
class
order
family
genus
species
10
Apicomplexa
Coccidia
Aconoidasida
Gregarinasina
Eucoccidiorida
Eugregarinorida
Neogregarinorida
Agamococcidiorida
Lecudinidae
Hepatozoidae
Haemogregarinidae
Lankesterellidae
Eimeriidae
Adeleidae
Babesiidae
Theileriidae
Cryptosporidiidae
Ophryocystidae
Rhytidocystidae
Stylocephalidae
Actinocephalidae
Sarcocystidae
Lankesteria
Veloxidium
Hepatozoon
Haemogregarina
Klossia
Toxoplasma
Goussia
Eimeria
Adelina
Schellackia
Babesia
Theileria
Cryptosporidium
Geneiorhynchus
Colepismatophila
Cardiosporidium
Rhytidocystis
Ophryocystis
Isospora
Nematopsis
Nephromyces
Hoplorhynchus
Neospora

Apicomplexa C primers amplicon length

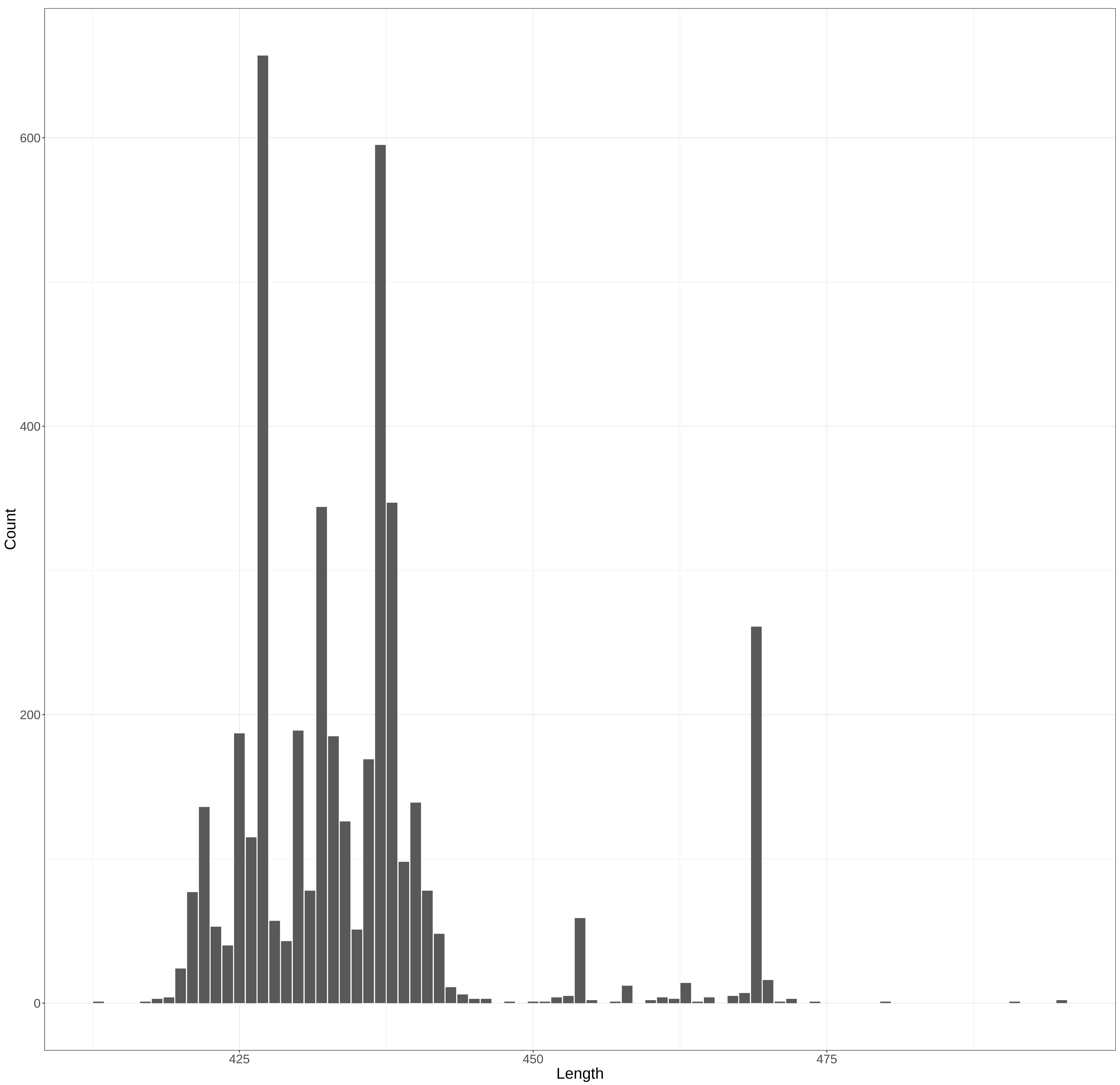

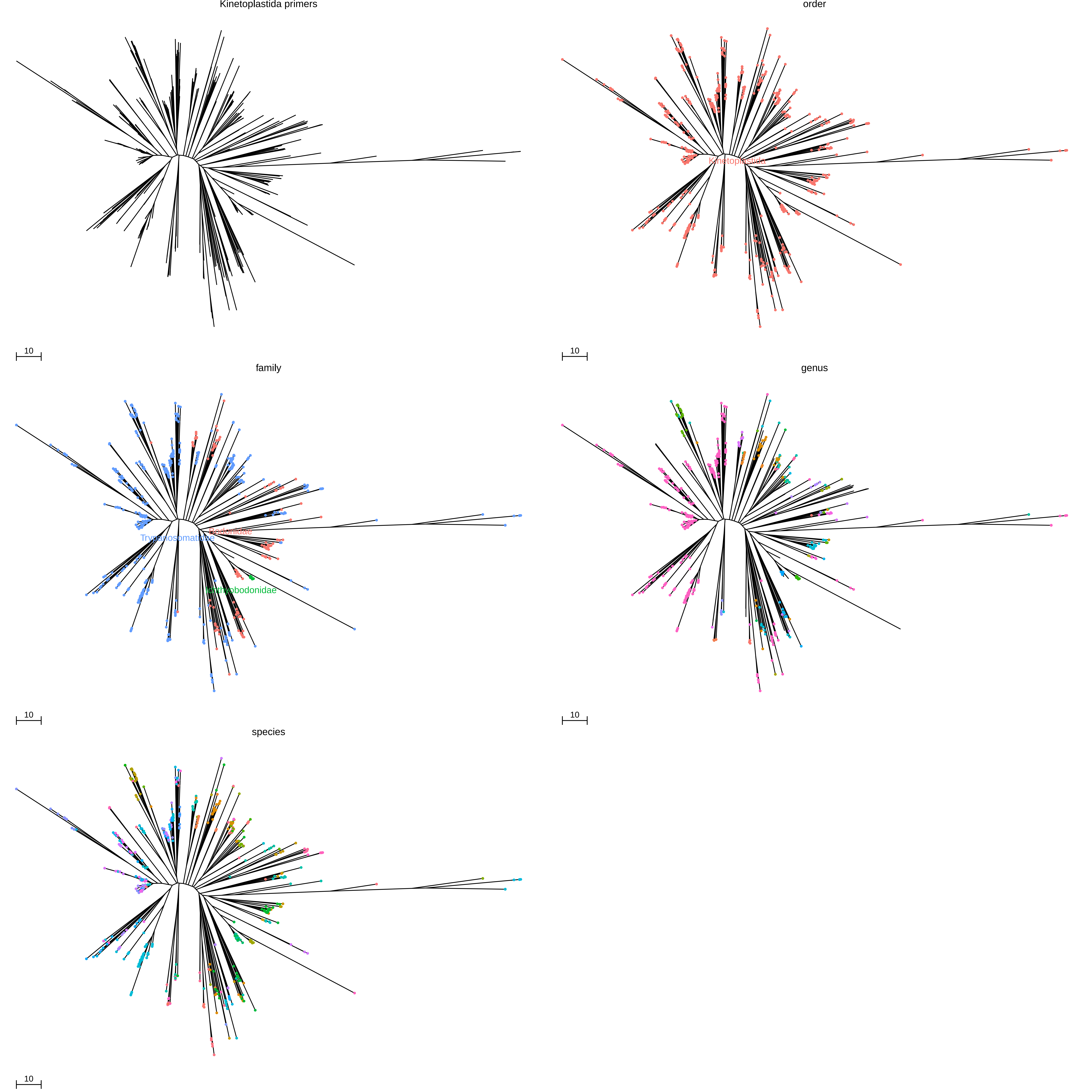
Kinetoplastida primers
order
Kinetoplastida
family
Trypanosomatidae
Bodonidae
genus
species
10

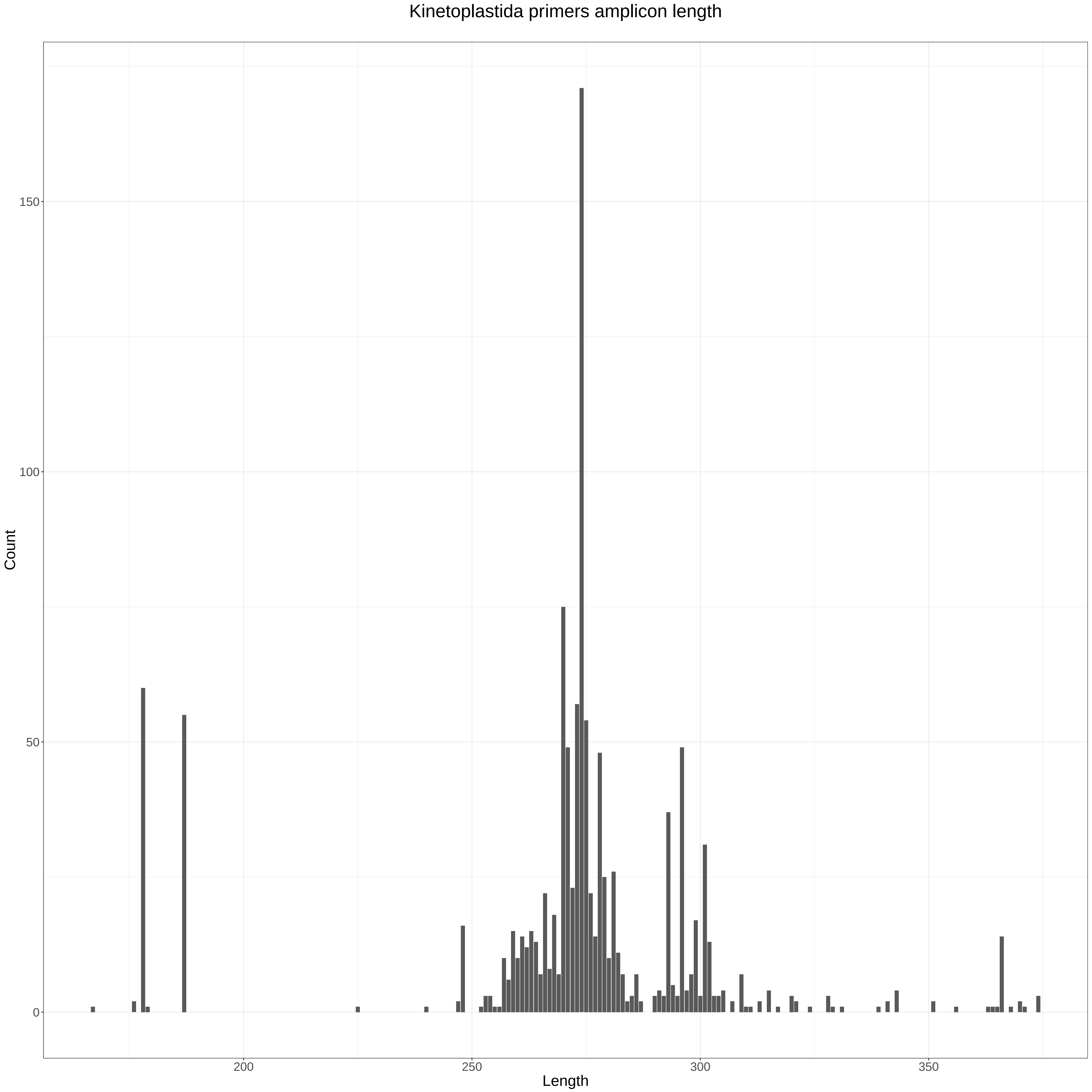
Kinetoplastida primers amplicon length
Count
150
100
50
0
200
250
300
350
Length

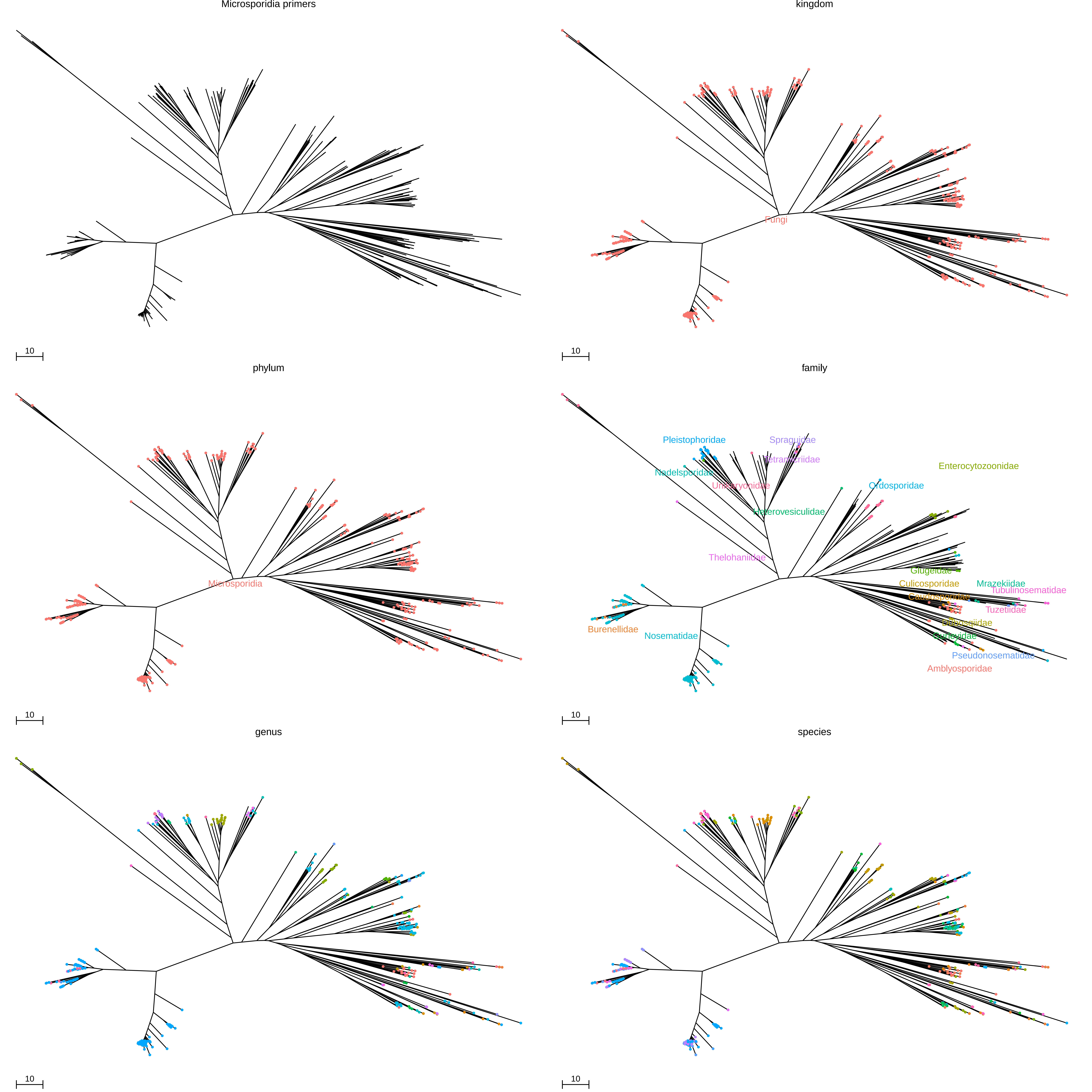

Microsporidia primers
10
kingdom
Fungi
10
phylum
Microsporidia
10
family
Pleistophoridae
Spraguidae
Tetramicriidae
Nadelsporidae
Unikaryonidae
Enterocytozoonidae
Ordosporidae
Heterovesiculidae
Thelohaniidae
Glugeidae
Culicosporidae
Mrazekiidae
Tubulinosematidae
Caudosporidae
Tuzetiidae
Dubosqiidae
Gurleyidae
Pseudonosematidae
Amblyosporidae
Burenellidae
Nosematidae
10
genus
10
species
10

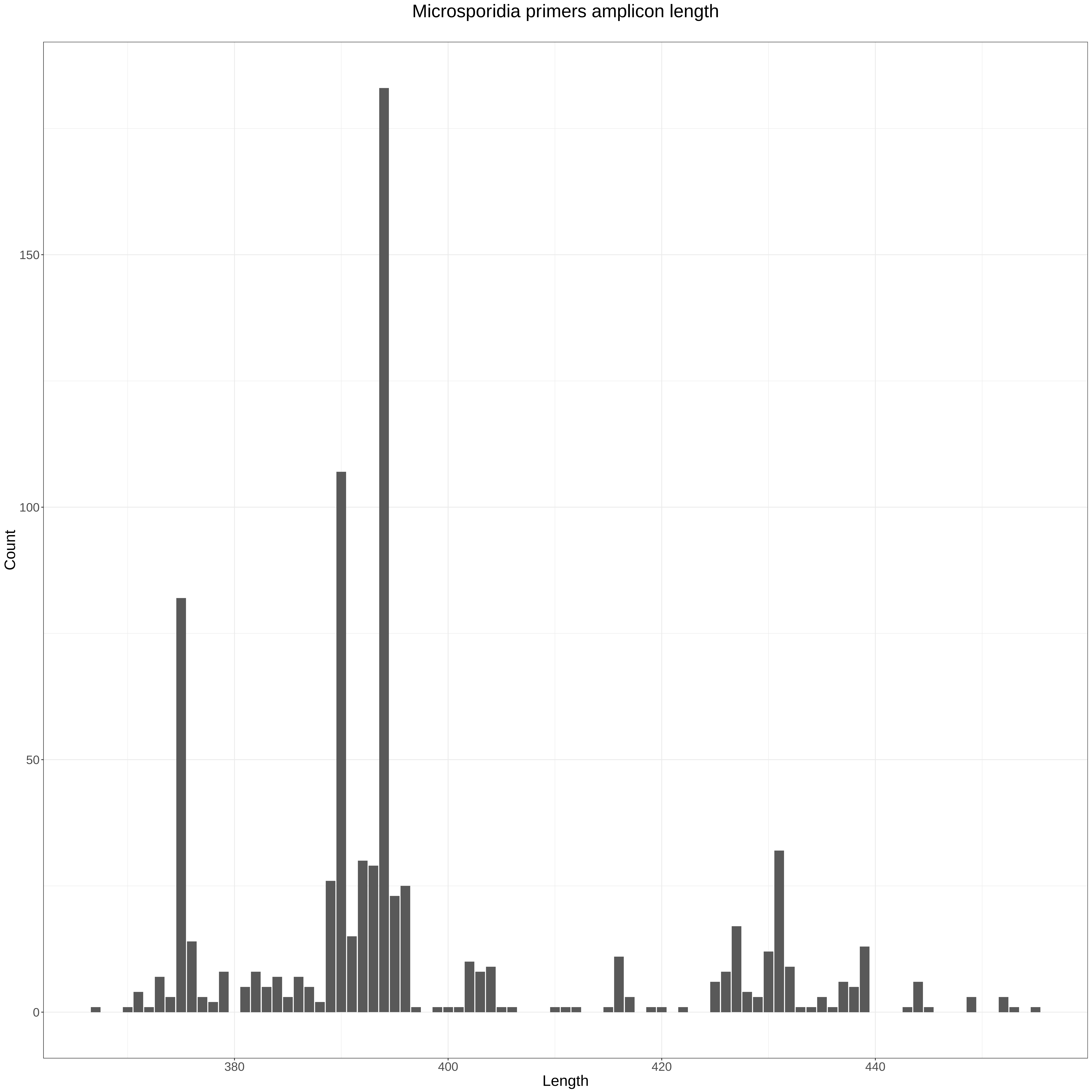
Microsporidia primers amplicon length
Count
150
100
50
0
380
400
420
440
Length

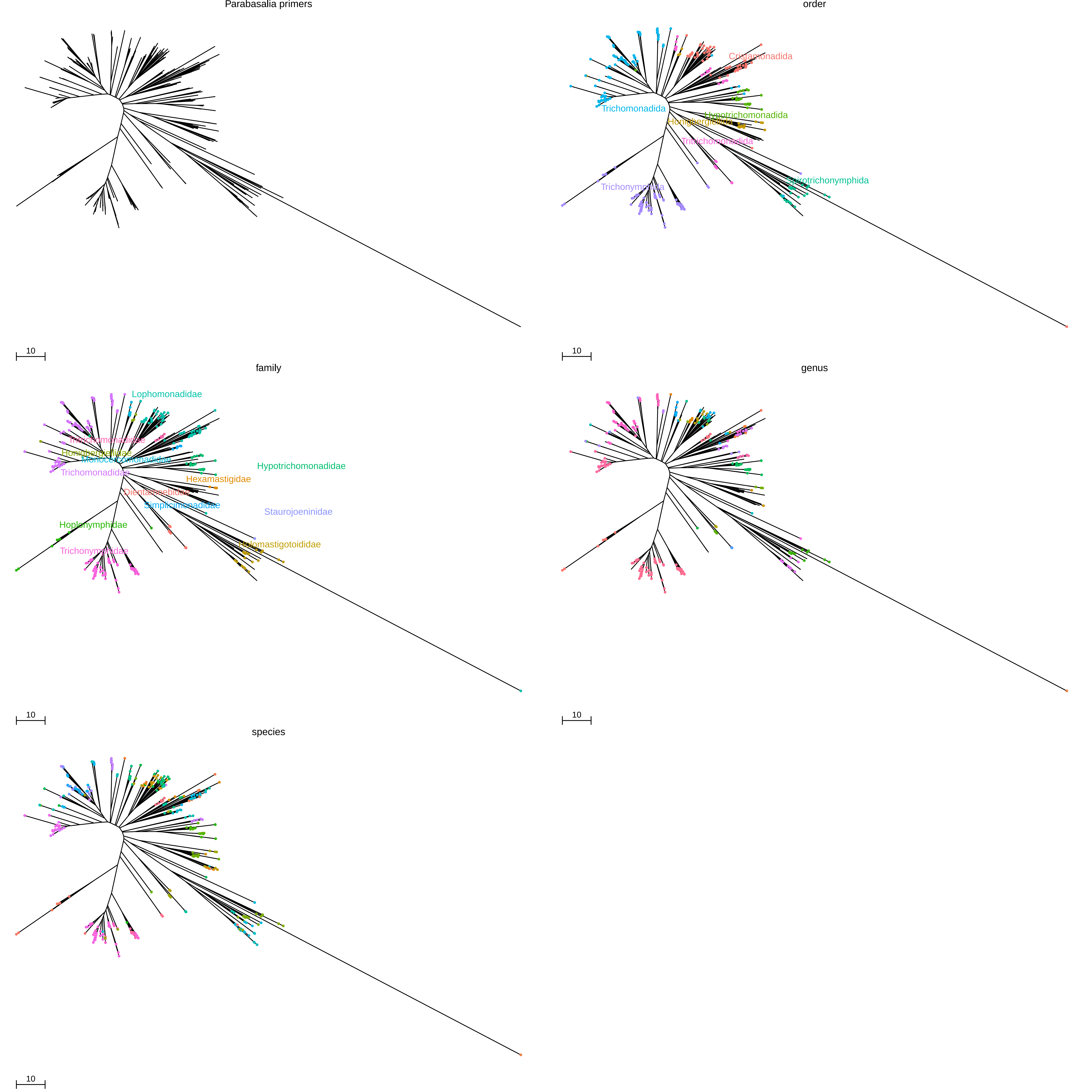
Parabasalia primers
10
order
Cristamonadida
Trichomonadida
Hypotrichomonadida
Honigbergiellida
Tritrichomonadida
Spirotrichonymphida
Trichonymphida
10
family
Lophomonadidae
Tritrichomonadidae
Honigbergiellidae
Monocercomonadidae
Trichomonadidae
Hypotrichomonadidae
Hexamastigidae
Dientamoebidae
Simplicimonadidae
Staurojoeninidae
Hoplonymphidae
Trichonymphidae
Holomastigotoididae
10
genus
10
species
10

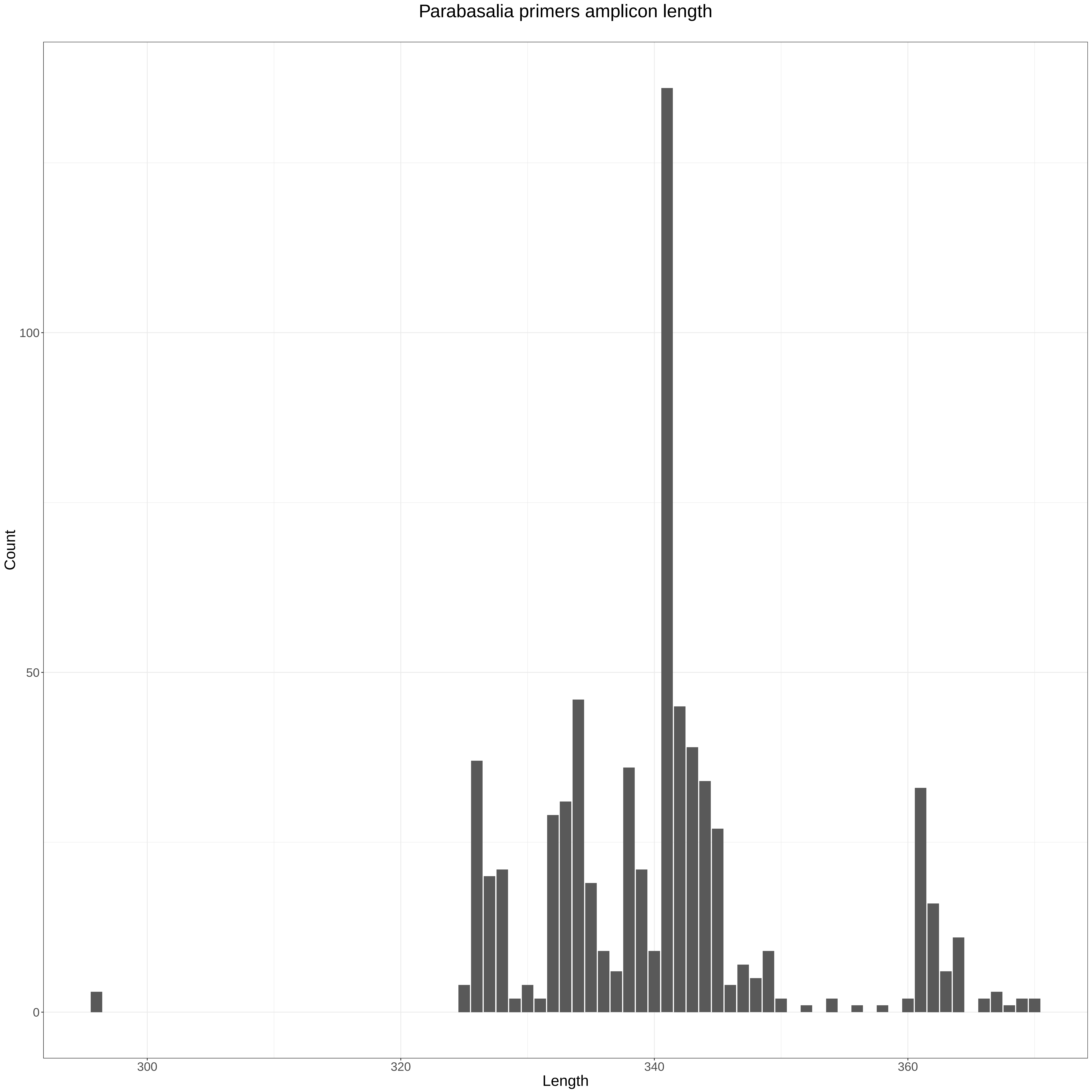
Parabasalia primers amplicon length
Count
100
50
0
300
320
340
360
Length

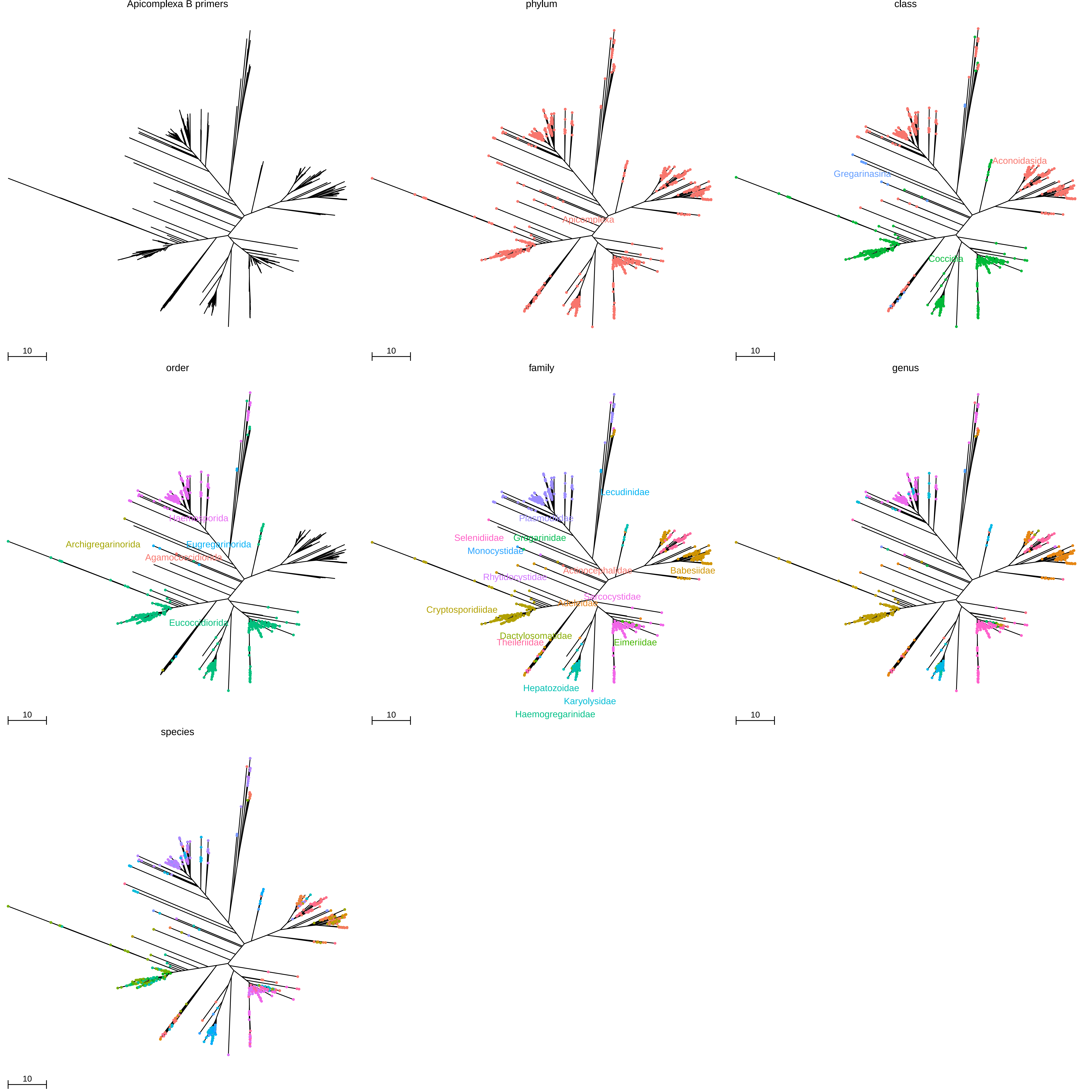

Apicomplexa B primers
10
phylum
Apicomplexa
10
class
Aconoidasida
Gregarinasina
Coccidia
10
order
Haemosporida
Archigregarinorida
Eugregarinorida
Agamococcidiorida
Eucoccidiorida
10
family
Lecudinidae
Plasmodiidae
Selenidiidae
Gregarinidae
Monocystidae
Rhytidocystidae
Actinocephalidae
Babesiidae
Sarcocystidae
Adeleidae
Cryptosporidiidae
Dactylosomatidae
Theileriidae
Eimeriidae
Hepatozoidae
Karyolysidae
Haemogregarinidae
10
genus
10
species
10

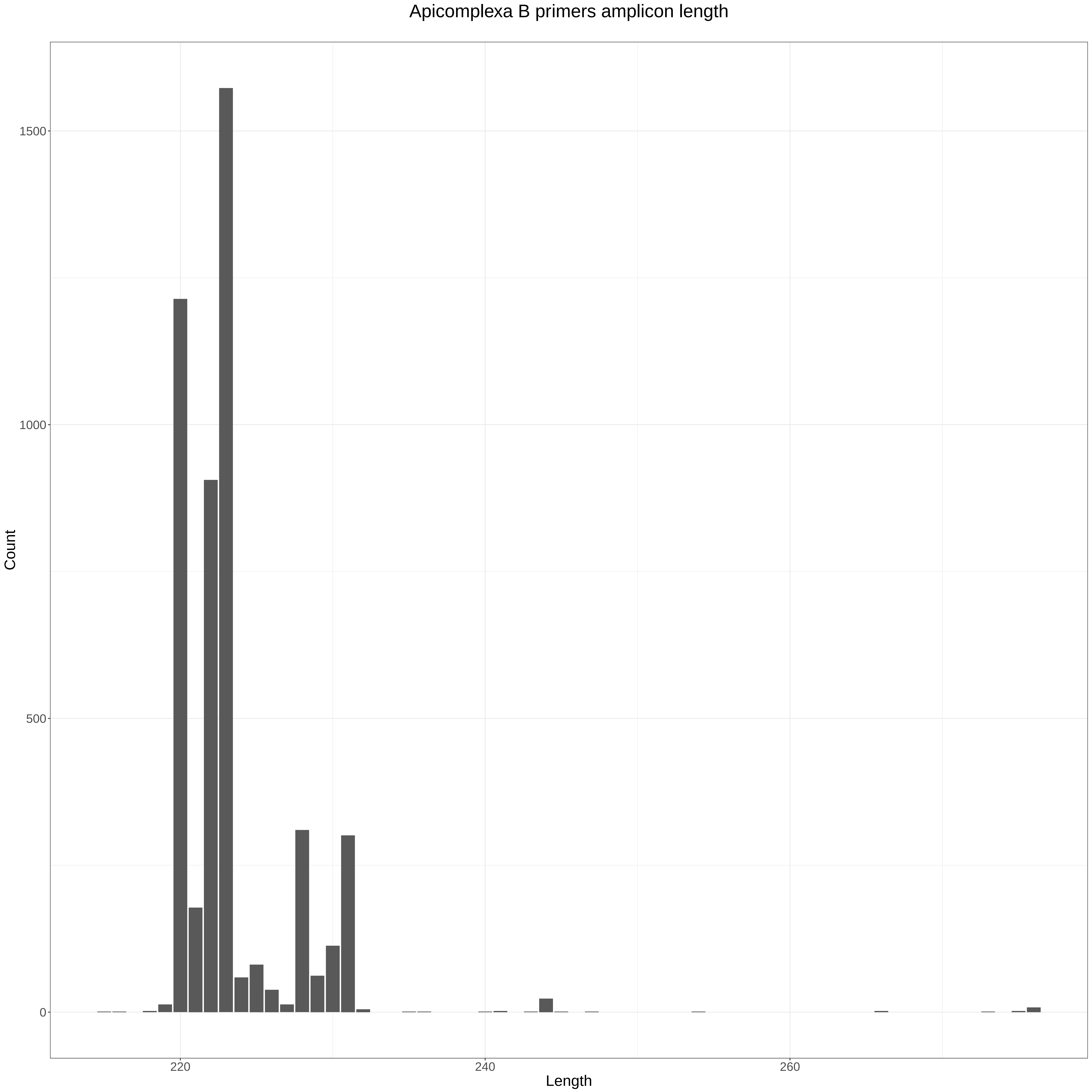
Apicomplexa B primers amplicon length
Count
1500
1000
500
0
220
240
260
Length

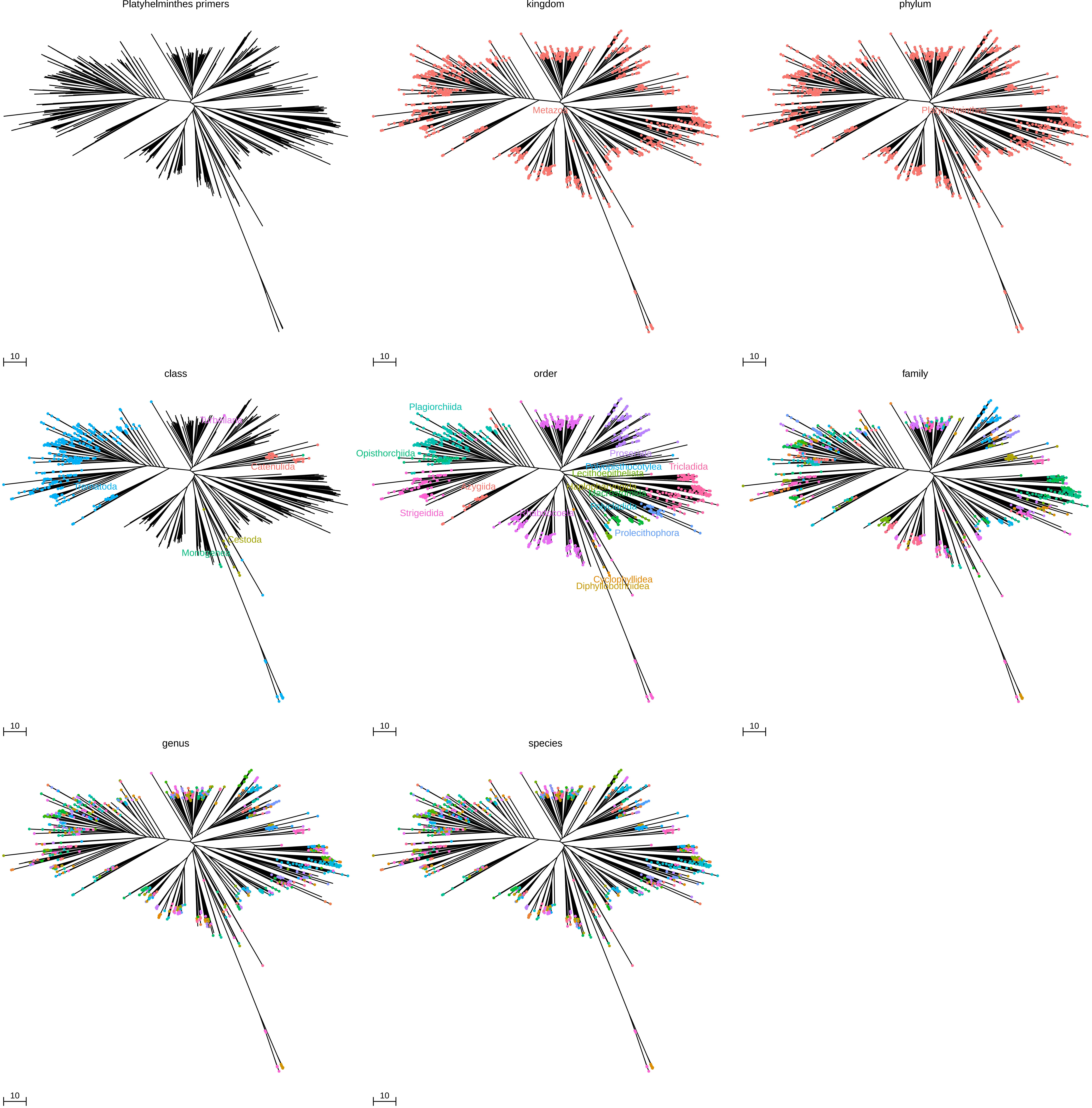

Platyhelminthes primers
kingdom
phylum
Metazoa
Platyhelminthes
class
Turbellaria
Catenulida
Trematoda
Cestoda
Monogenea
order
Plagiorchiida
Opisthorchiida
Proseriata
Polyopisthocotylea
Tricladida
Lecithoepitheliata
Azygiida
Haplopharyngida
Macrostomida
Strigeidida
Rhabdocoela
Polycladida
Prolecithophora
Cyclophyllidea
Diphyllobothriidea
family
genus
species
10

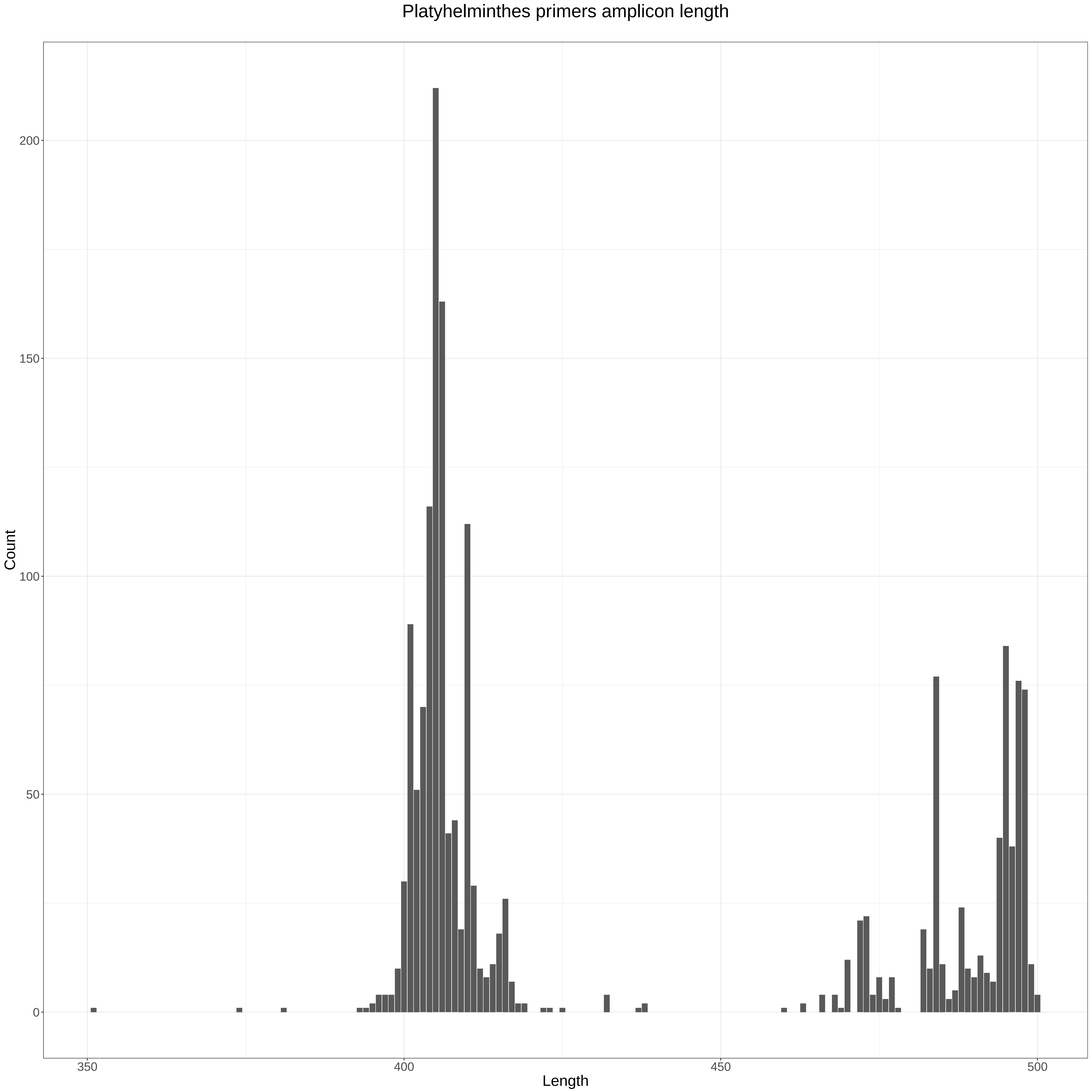

Platyhelminthes primers amplicon length
Count
Length
0
50
100
150
200
350
400
450
500

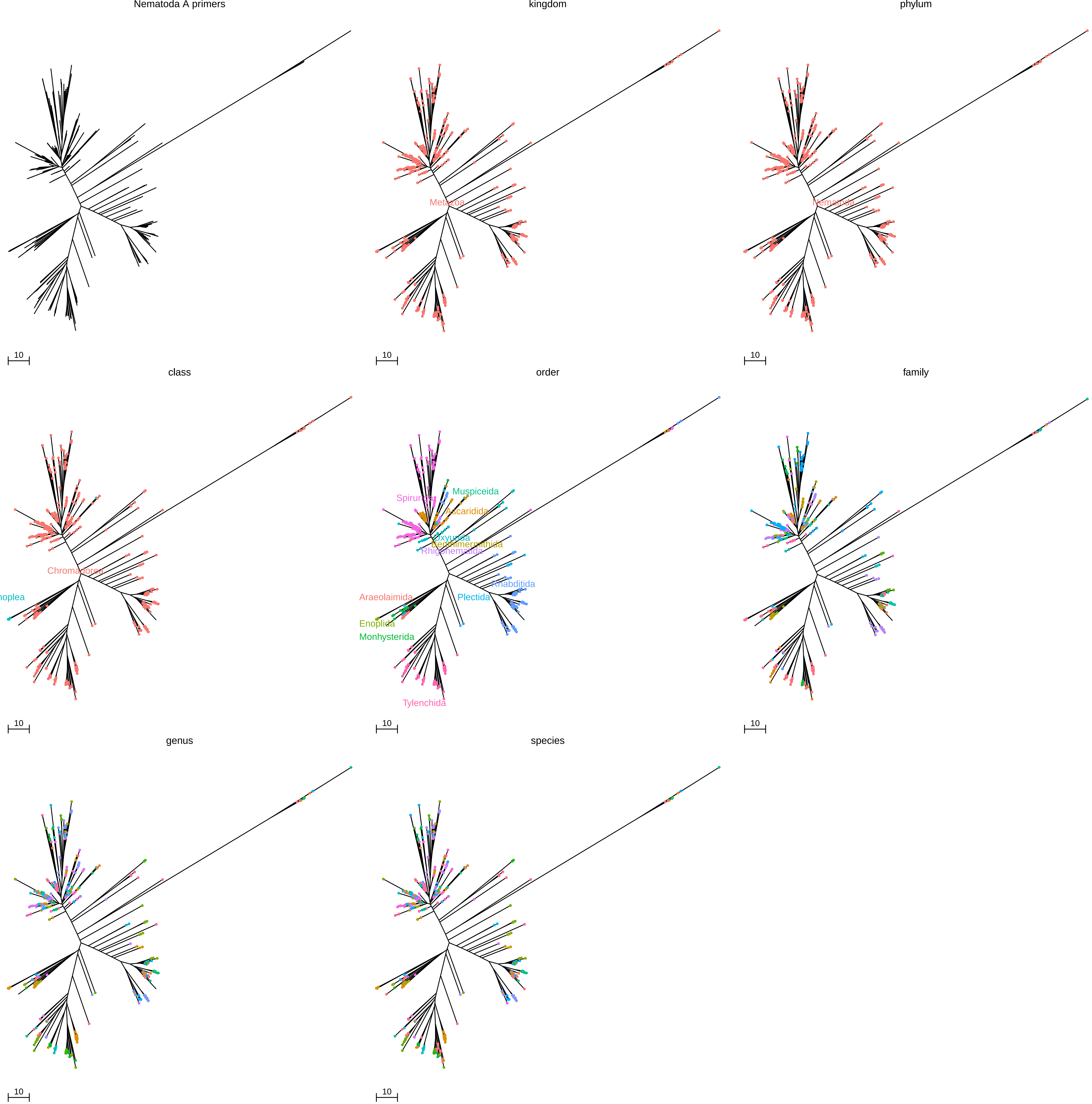
Nematoda A primers
10
kingdom
Metazoa
10
phylum
Nematoda
10
class
Chromadorea
noplea
10
order
Spirurida
Muspiceida
Ascaridida
Oxyurida
Benthimermithida
Rhigonematida
Rhabditida
Araeolaimida
Plectida
Enoplida
Monhysterida
Tylenchida
10
family
10
genus
10
species
10

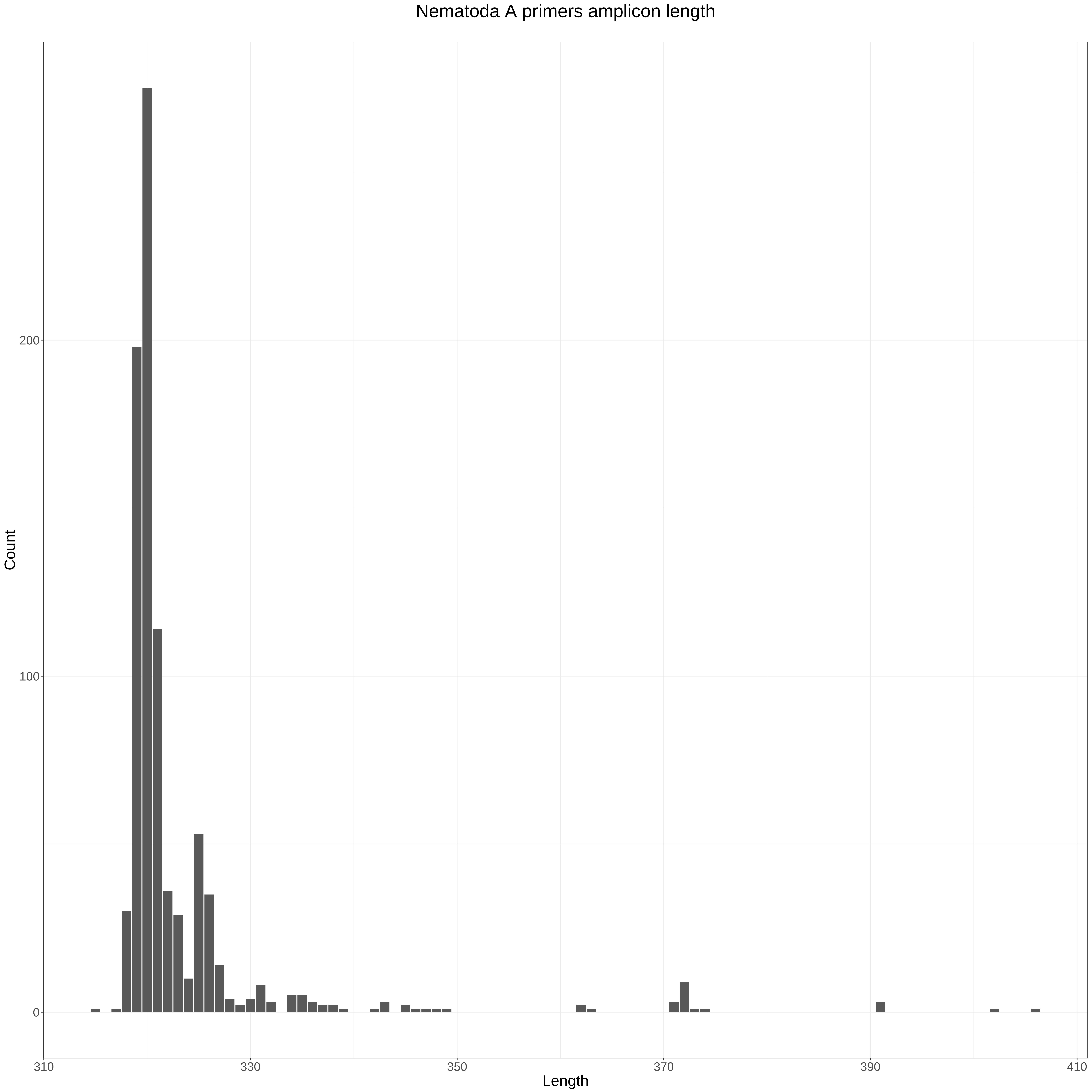
Nematoda A primers amplicon length
Count
Length
200
100
0
310
330
350
370
390
410

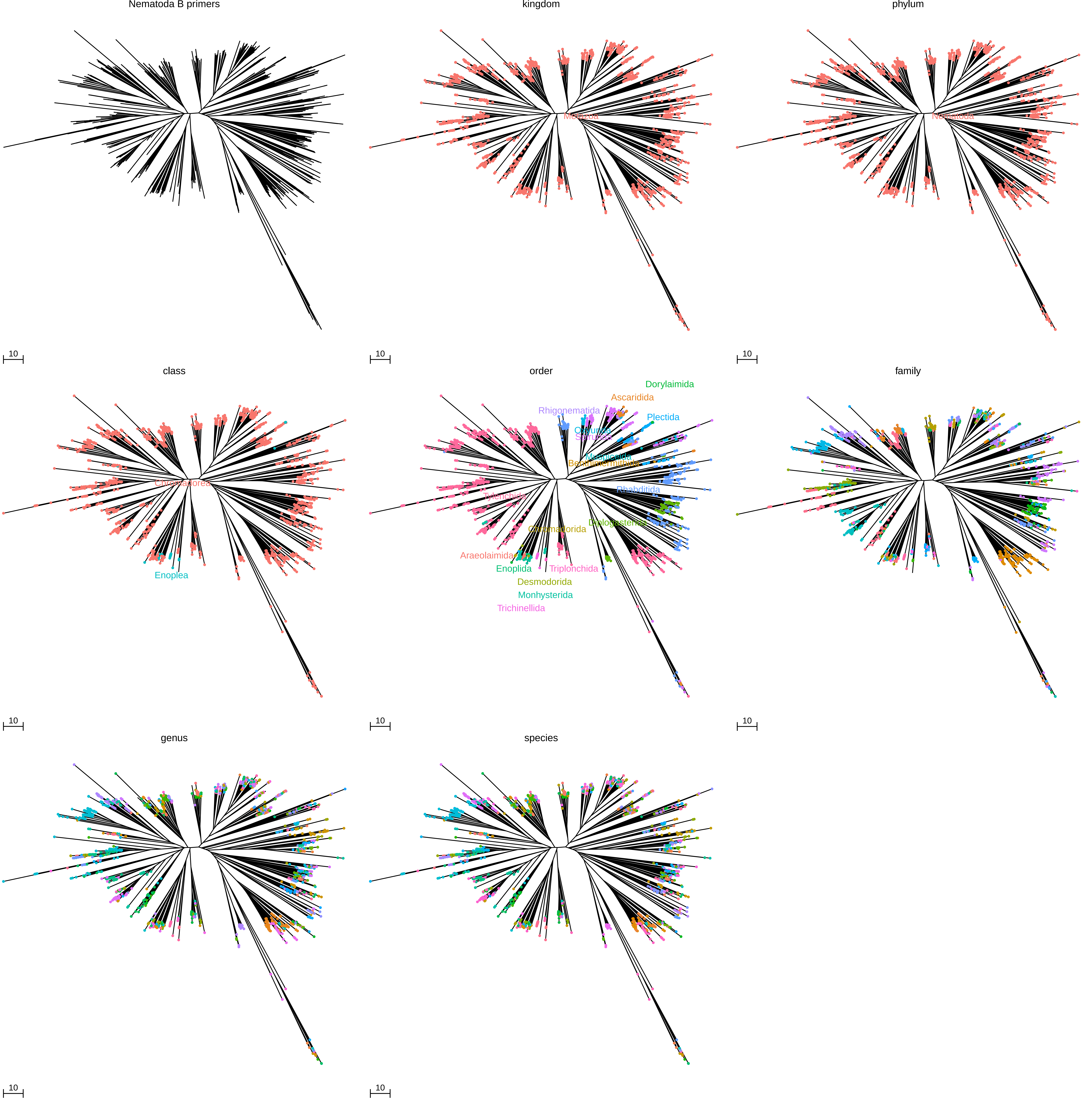
Nematoda B primers
kingdom
phylum
Nematoda
class
Chromadorea
Enoplea
order
Dorylaimida
Ascaridida
Rhigonematida
Plectida
Benthimermithida
Rhabditida
Tylenchida
Diplogasterida
Chromadorida
Araeolaimida
Enoplida
Triplonchida
Desmodorida
Monhysterida
Trichinellida
family
genus
species
10

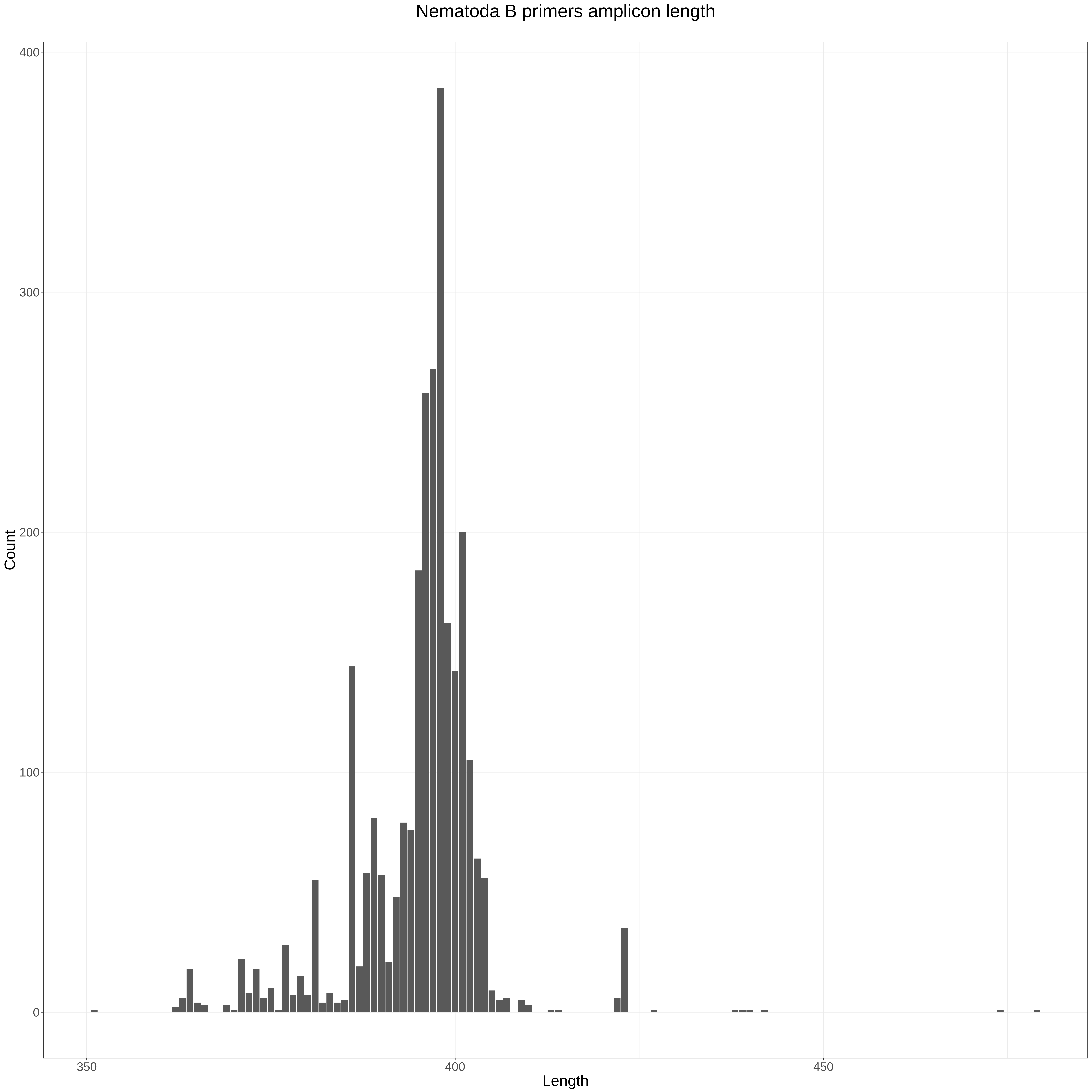
Nematoda B primers amplicon length
Count
Length
0
100
200
300
400
350
400
450

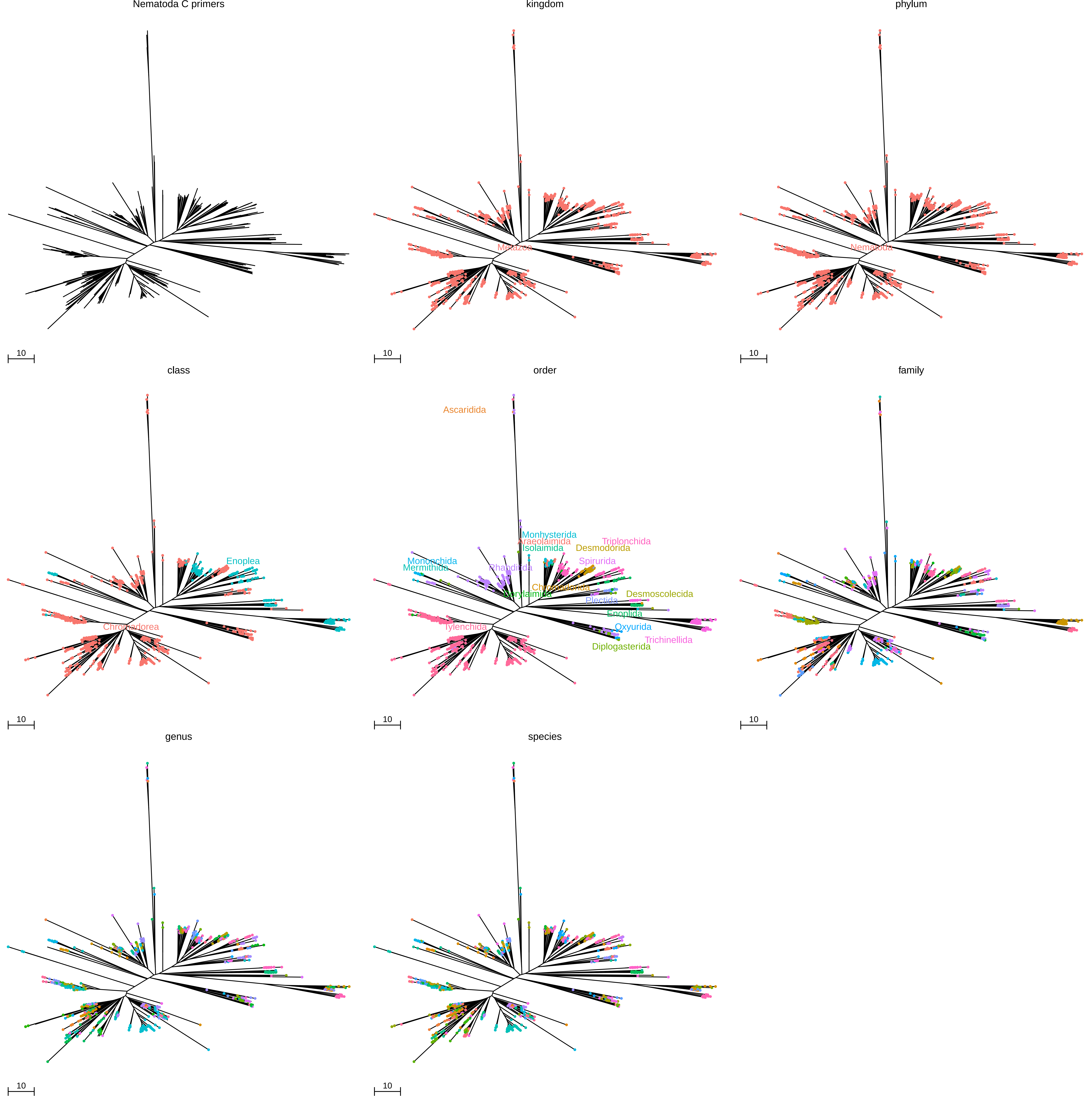

Nematoda C primers
kingdom
phylum
10
10
10
Metazoa
Nematoda
class
order
family
Enoplea
Chromadorea
Ascaridida
Monhysterida
Araeolaimida
Isolaimida
Triplonchida
Desmodorida
Mononchida
Mermithida
Rhabditida
Spirurida
Chromadorida
Dorylaimida
Desmoscolecida
Plectida
Enoplida
Oxyurida
Tylenchida
Trichinellida
Diplogasterida
10
10
10
genus
species
10
10

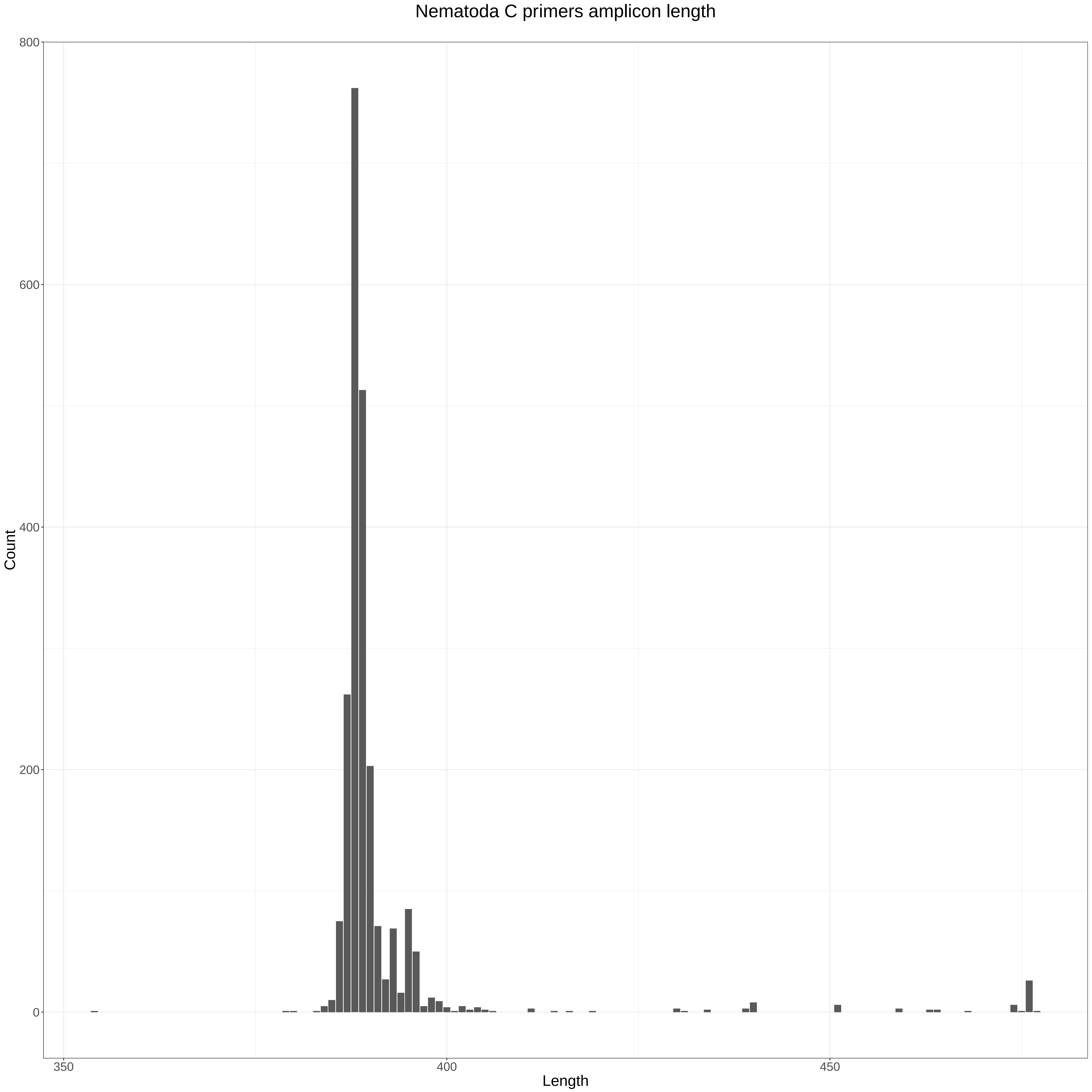
Nematoda C primers amplicon length
Count
Length
800
600
400
200
0
350
400
450
