# Supplementary material for: A high-throughput sequencing assay to comprehensively detect and characterize unicellular eukaryotes and helminths from biological and environmental samples
Source: Microbiome. 2018 Oct 29;6:195. doi: 10.1186/s40168-018-0581-6 (PMC6206884; doi:10.1186/s40168-018-0581-6)
Supplement: Supplementary file 1 — Table S1. Examples of genera targeted by each primer pair. Table S2. Primer characteristics of primers from the literature. Table S3. Amplification of positive controls. The table shows the results of the sequencing assay when performed on pools of DNA from known parasites, with and without addition of Anopheles/Escherichia/Human DNA. Each column shows the percentage of the reads that match each observed species. Green text represents on-target species and red text shows off-target (often host) species amplification. Table S4. Results from all samples. Explanation of each column is presented in the excel sheet. Figure S1. Complementarity of the primer pairs targeting the same taxonomic groups. Apicomplexa and Nematoda each required three primer pairs to capture the diversity within these groups. The taxa amplified by each of the three primer sets are presented as Venn diagrams showing the overlap in species coverage. The percent within each sector is shown in parentheses. Figure S2. Overview of the pipeline for the in silico assessment of the primer amplification range, information content and specificity. Figure S3. PrimerTree results for each newly designed primer. The figures show, for each primer set, the PrimerTree plot and amplicon lengths. The PrimerTree results were restricted only to the targeted group to show the diversity of on-target taxonomic groups amplifiable. (ZIP 2497 kb) [file 40168_2018_581_MOESM1_ESM.zip › SupplementalMaterial_Final.docx]

**Additional file TABLE AND FIGURE LEGENDS**

| Taxon Targeted | Examples of some of the genera targeted |
| --- | --- |
| Amoebozoa | *Acanthamoeba, Dictyostelium, Endolimax, Entamoeba, Heterostelium, Lamproderma, Physarum* |
| Apicomplexa A | *Babesia, Cryptosporidium, Cyclospora, Eimeria, Hepatozoon, Isospora, Plasmodium, Sarcocystis, Theileria, Toxoplasma* |
| Apicomplexa B |  |
| Apicomplexa C |  |
| Blastocystis | *Blastocystis* |
| Diplomonadida | *Giardia, Hexamita, Spironucleus* |
| Kinetoplastida | *Bodo, Crithidia, Herpetomonas, Ichthyobodo, Leishmania, Leptomonas, Trypanosoma* |
| Microsporidia | *Amblyospora, Dictyocoela, Encephalitozoon, Ichthyosporidium, Leibermannia, Nosema, Vairimorpha* |
| Nematode A | *Ancylostoma, Ascaris, Brugia, Dracunculus, Loa, Mansonella, Onchocerca, Trichinella, Trichuris, Wuchereria* |
| Nematode B |  |
| Nematode C |  |
| Parabasalia | *Coronympha, Hypotrichomonas, Tetratrichomonas, Trichomonas, Tritrichomonas* |
| Platyhelminthes | *Diphyllobothrium, Echinococcus, Gyrodactylus, Macrostomum, Raillietina, Schistosoma, Taenia, Trichobilharzia* |

**Table S1 Examples of genera targeted by each primer pair.**

| Primer name | Amplicon Length (in bp) | No. species amplifiable (%NCBI*) | Matching single genus (mean) | Matching single species (mean) | Specificity (%on target) | Forward primer | Reverse primer | Citation |
| --- | --- | --- | --- | --- | --- | --- | --- | --- |
| Amoebozoa_Ami6F1_Ami9R | 500-1,000 | 338 (99.4%) | 1 (99.4%) | 1.1 (92.8%) | 0.1% | CCAGCTCCAATAGCGTATATT | GTTGAGTCGAATTAAGCCGC | [1] |
| Ciliophora_121F_1147R | 795-900 | 1,129 (91%) | 1 (97%) | 1.1 (92.6%) | 59.6% | CTGCGAATGGCTCATTAMAA | GACGGTATCTRATCGTCTTT | [2] |
| Dinophyceae_18ScomF1_Dino18SR1 | 645-660 | 261 (68.9%) | 1 (98.4%) | 1.1 (92.4%) | 98.4% | GCTTGTCTCAAAGATTAAGCCATGC | GAGCCAGATRCDCACCCA | [3] |
| Diplomonadida_DimA_DimB | 1,420-1,425 | 2 (66.7%) | 1 (100%) | 1 (100%) | 0.0% | AACCTGGTTGATCTTGCCAG | CYGCAGGTTCACCTACGGAA | [4] |
| Entamoeba_JVF_DSPR2 | 530-640 | 17 (81%) | 1 (100%) | 1.2 (75.7%) | 5.6% | GTTGATCCTGCCAGTATTATATG | CACTATTGGAGCTGGAATTAC | [5] |
| Eukaryota_E528F_Univ1391RE | 1,020-1,350 | NA** | 1.1 (95.8%) | 1.3 (90.5%) | 99.9% | CGGTAATTCCAGCTCC | ACCTTGTTACGRCTT | [6, 7] |
| Eukaryota_E528F_Univ1492RE | 870-1,285 | NA** | 1 (98.5%) | 1.1 (94.5%) | 99.9% | CGGTAATTCCAGCTCC | GGGCGGTGTGTACAARGRG | [6, 7] |
| Eukaryota_EK-82F_EK-1520 | 1,495-1,870 | NA** | 1.1 (95.8%) | 1.5 (89.8%) | 99.9% | GAAACTGCGAATGGCTC | CYGCAGGTTCACCTAC | [8] |
| Eukaryota_Euk1A_Euk516r | 500-615 | NA** | 1.8 (87.4%) | 3.3 (77.5%) | 100.0% | CTGGTTGATCCTGCCAG | ACCAGACTTGCCCTCC | [9, 10] |
| Eukaryota_EUKA_EUKB | 1,635-1,925 | NA** | 1 (97.2%) | 1.2 (92.4%) | 100.0% | AACCTGGTTGATCCTGCCAGT | TGATCCTTCTGCAGGTTCACCTAC | [11] |
| Eukaryota_F-566_R-1200 | 590-865 | NA** | 1.4 (89.1%) | 2 (80.3%) | 100.0% | CAGCAGCCGCGGTAATTCC | CCCGTGTTGAGTCAAATTAAGC | [12] |
| Eukaryota_FUNF_FUNR | 995-1,040 | NA** | 1.1 (93.5%) | 1.3 (83.8%) | 99.8% | GATCCCTAGTCGGCATAGTT | GTAGTCATATGCTTGTCTC | [13] |
| Eukaryota_NSI_FR1 | 1,455-1,690 | NA** | 1.1 (91.8%) | 1.4 (81.9%) | 99.9% | GTAGTCATATGCTTGTCTC | AICCATTCAATCGGTAIT | [14, 15] |
| Eukaryota_TAReuk454FWD1_TAReukREV3 | 345-685 | NA** | 2.1 (86.4%) | 2.9 (77.3%) | 99.9% | CCAGCASCYGCGGTAATTCC | ACTTTCGTTCTTGATYRA | [16] |
| Eukaryota_Uni18SF_Uni18SR | 405-535 | NA** | 2.1 (85.5%) | 3 (73.1%) | 99.9% | AGGGCAAKYCTGGTGCCAGC | GRCGGTATCTRATCGYCTT | [17] |
| Fungi_ITSF_ITS-4R | 395-805 | NA** | 1 (98.6%) | 1.2 (92.6%) | 97.1% | CTTGGTCATTTAGAGGAAGTAA | TCCTCCGCTTATTGATATGC | [18, 19] |
| Kinetoplastidia_Kineto_kin1_Kineto_kin2 | 405-535 | 27 (79.4%) | 1 (100%) | 1 (97.1%) | 90.9% | GCGTTCAAAGATTGGGCAAT | CGCCCGAAAGTTCACC | [20] |
| Microsporidia_V1_PMP2 | 240-300 | 59 (36.9%) | 1 (97.9%) | 1 (95.8%) | 97.7% | CACCAGGTTGATTCTGCCTGAC | CCTCTCCGGAACCAAACCCTG | [21] |
| Trichomonadida_TFR1_TFR2 | 365-375 | 10 (83.3%) | 1 (100%) | 1.1 (94.7%) | 21.7% | TGCTTCAGTTCAGCGGGTCTTCC | CGGTAGGTGAACCTGCCGTTGG | [22] |

**Table S2. Primer characteristics of primers from the literature.**

The table shows for each primer pair, expected amplicon lengths and a summary of the *in silico* assessment of the primer amplification range, information content and specificity. It notably indicates the number of species amplified *in silico* (and the proportion of species deposited in NCBI that this represents), the proportion of DNA sequences that matched a single genus/species (and the mean number of genera/species matching each DNA sequence), and the proportion of amplified DNA sequences belonging to the targeted taxon. The last two columns contain the primer sequences. **General Eukaryote primers did not have this metric calculated because the PrimerTree analysis allowed only 10,000 hits at once, which results in an underestimation of this metric due to the high number of Eukaryotes.

| **Primer and species amplified** | **Parasite + host rep. 1** | **Parasite + host rep. 2** | **Parasite rep. 2** | **Parasite rep. 1** |
| --- | --- | --- | --- | --- |
| **Amoebozoa** |  |  |  |  |
| Anopheles gambiae | 72.53% | 0.00% | 0.00% | 0.00% |
| Dictyostelium discoideum | 15.18% | 0.00% | 0.00% | 50.64% |
| Saccharomycetes fungus sp. | 7.87% | 0.00% | 0.00% | 29.65% |
| Schistosoma sp. | 2.66% | 0.00% | 0.00% | 10.36% |
| Rhizopus sp. | 0.93% | 0.00% | 0.00% | 6.45% |
| Theileria parva | 0.53% | 0.00% | 0.00% | 1.92% |
| Unknown sequence | 0.29% | 0.00% | 0.00% | 0.00% |
| Cryptosporidium sp. | 0.00% | 0.00% | 0.00% | 0.68% |
| Acanthocheilonema viteae | 0.00% | 0.00% | 0.00% | 0.31% |
| **Diplomonadida** |  |  |  |  |
| Giardia intestinalis | 99.94% | 100.00% | 100.00% | 100.00% |
| Homo sapiens | 0.06% | 0.00% | 0.00% | 0.00% |
| **Apicomplexa C** |  |  |  |  |
| Theileria parva | 89.61% | 83.33% | 97.21% | 95.99% |
| Homo sapiens | 8.96% | 16.67% | 0.00% | 1.23% |
| Cryptosporidium sp. | 1.44% | 0.00% | 2.79% | 1.84% |
| Leishmania major strain Friedlin | 0.00% | 0.00% | 0.00% | 0.73% |
| Cryptosporidium parvum | 0.00% | 0.00% | 0.00% | 0.20% |
| **Kinetoplastida** |  |  |  |  |
| Leishmania sp. | 97.59% | 97.85% | 97.69% | 97.39% |
| Trypansoma sp. | 2.41% | 2.15% | 2.31% | 2.61% |
| Trypanosoma brucei rhodesiense | 0.20% | 0.17% | 0.21% | 0.24% |
| **Microsporidia** |  |  |  |  |
| Encephalitozoon cuniculi/Encephalitozoon cuniculi GB-M1 | 99.01% | 100.00% | 100.00% | 99.35% |
| Encephalitozoon cuniculi | 0.99% | 0.00% | 0.00% | 0.65% |
| **Apicomplexa B** |  | 0.09% |  |  |
| Escherichia coli | 84.94% | 100.00% | 0.00% | 0.00% |
| Homo sapiens | 15.06% | 0.00% | 0.00% | 0.00% |
| Rhizopus sp. | 0.00% | 0.00% | 0.00% | 51.48% |
| Cryptosporidium sp. | 0.00% | 0.00% | 0.00% | 48.52% |
| **Platyhelminthes** |  |  |  |  |
| Rhizopus sp. | 35.48% | 59.26% | 64.29% | 66.67% |
| Schistosoma mansoni/Schistosoma rodhaini | 64.52% | 40.74% | 35.71% | 33.33% |
| **Nematoda A** |  |  |  |  |
| Acanthocheilonema viteae | 99.73% | 99.74% | 99.85% | 99.86% |
| Acanthocheilonema odendhali | 0.27% | 0.26% | 0.15% | 0.14% |
| **Nematoda B** |  |  |  |  |
| Acanthocheilonema viteae | 100.00% | 0.00% | 100.00% | 100.00% |
| **Nematoda C** |  |  |  |  |
| Schistosoma sp. | 100.00% | 100.00% | 100.00% | 100.00% |

**Table S3.** **Amplification of positive controls.** The table shows the results of the sequencing assay when performed on pools of DNA from known parasites, with and without addition of *Anopheles/Escherichia*/Human DNA. Each column shows the percentage of the reads that match each observed species. Green text represents on-target species and red text shows off-target (often host) species amplification.

See Stable4.xlsx

**Table S4. Results from all samples. Explanation of each column is presented in the excel sheet.**


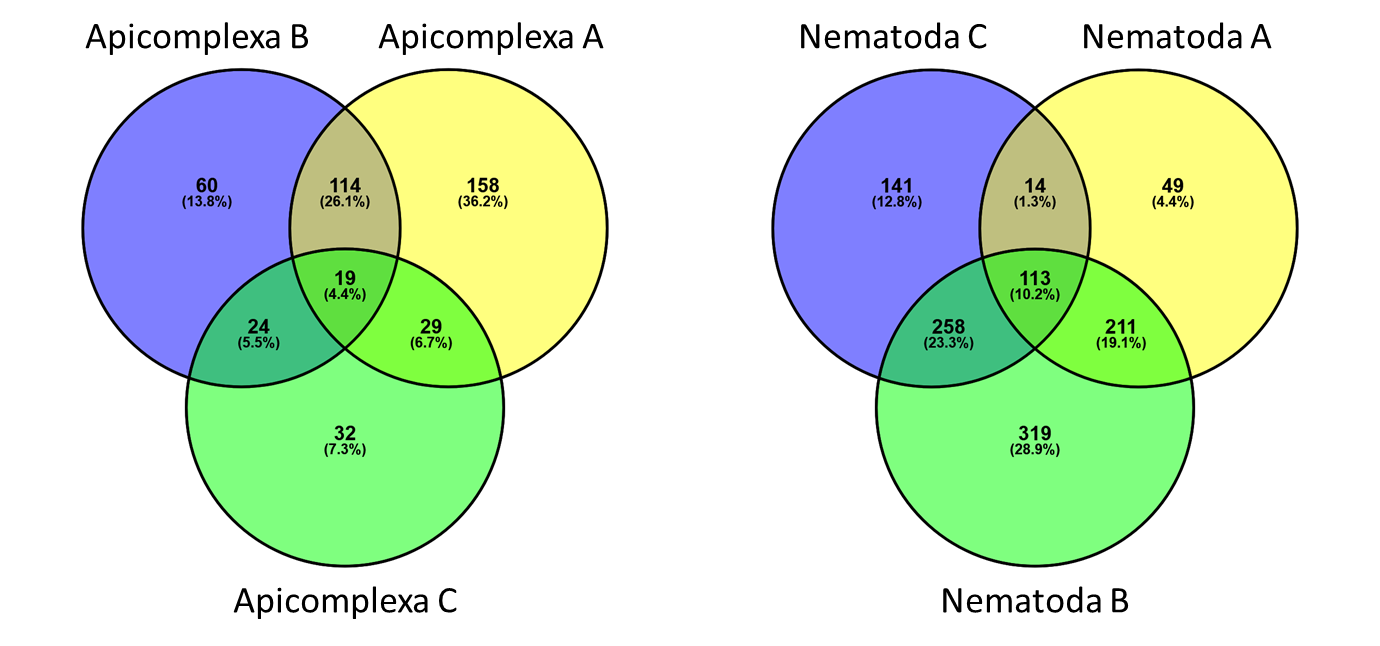


**Figure S1** **Complementarity of the primer pairs targeting the same taxonomic groups.** *Apicomplexa* and *Nematoda* each required three primer pairs to capture the diversity within these groups. The taxa amplified by each of the three primer sets are presented as Venn diagrams showing the overlap in species coverage. The percent within each sector is shown in parentheses.

**
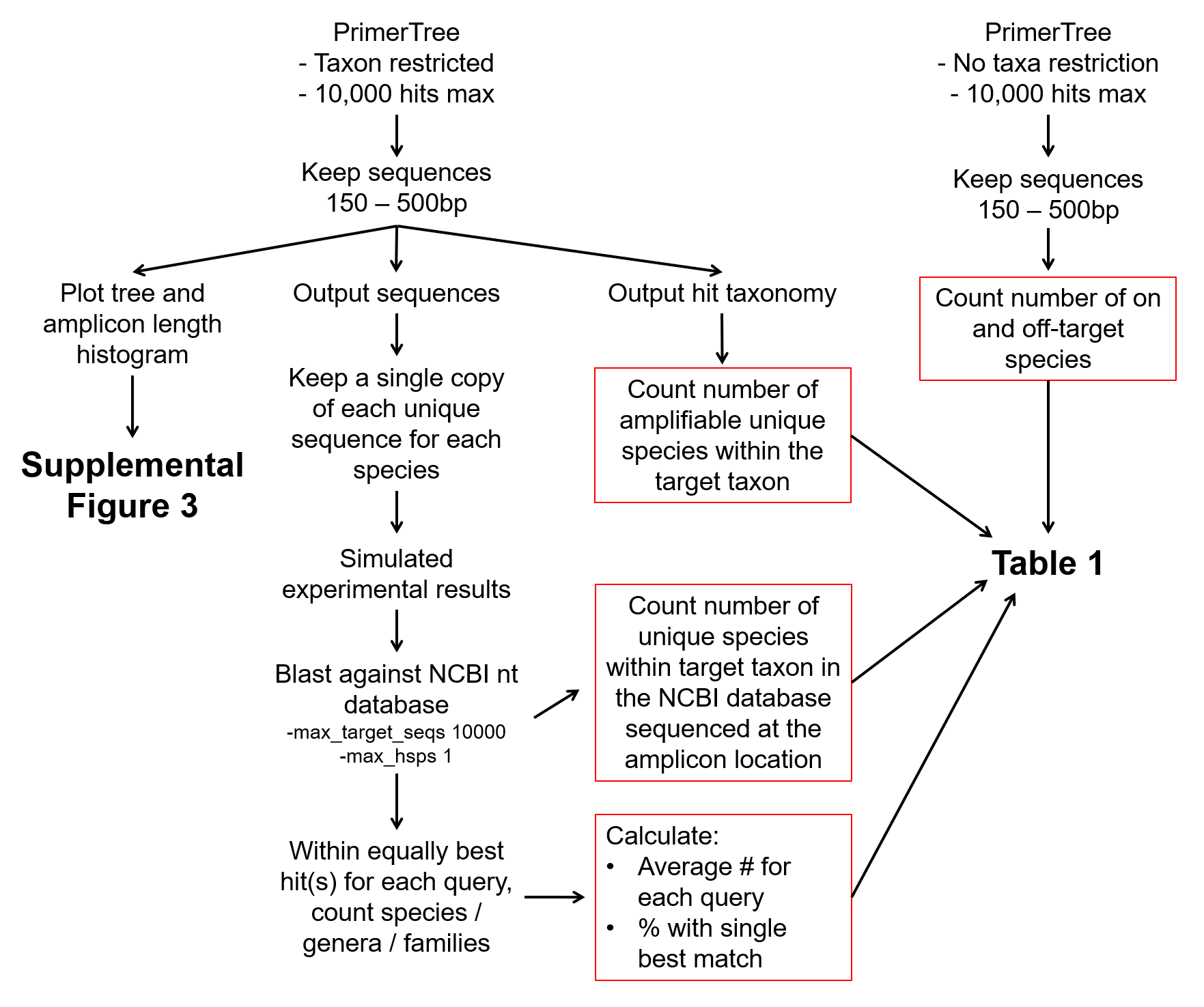
**

**Figure S2.** **Overview of the pipeline for the *in silico* assessment of the primer amplification range, information content and specificity.** Code available on https://github.com/MVesuviusC/2018_methods_paper.

See SFigure_3.pdf

**Figure S3.** **PrimerTree results for each newly designed primer.** The figures show, for each primer set, the PrimerTree plot and amplicon lengths. The PrimerTree results were restricted only to the targeted group to show the diversity of on-target taxonomic groups amplifiable.

Supplemental references:

1. Thomas V, Herrera-Rimann K, Blanc DS, Greub G: **Biodiversity of amoebae and amoeba-resisting bacteria in a hospital water network**. *Appl Environ Microbiol* 2006, **72**(4):2428-2438.

2. Dopheide A, Lear G, Stott R, Lewis G: **Molecular characterization of ciliate diversity in stream biofilms**. *Appl Environ Microbiol* 2008, **74**(6):1740-1747.

3. Lin S, Zhang H, Hou Y, Miranda L, Bhattacharya D: **Development of a dinoflagellate-oriented PCR primer set leads to detection of picoplanktonic dinoflagellates from Long Island Sound**. *Appl Environ Microbiol* 2006, **72**(8):5626-5630.

4. Kolisko M, Silberman JD, Cepicka I, Yubuki N, Takishita K, Yabuki A, Leander BS, Inouye I, Inagaki Y, Roger AJ *et al*: **A wide diversity of previously undetected free-living relatives of diplomonads isolated from marine/saline habitats**. *Environ Microbiol* 2010, **12**(10):2700-2710.

5. Verweij JJ, Laeijendecker D, Brienen EA, van Lieshout L, Polderman AM: **Detection and identification of entamoeba species in stool samples by a reverse line hybridization assay**. *J Clin Microbiol* 2003, **41**(11):5041-5045.

6. Edgcomb VP, Kysela DT, Teske A, de Vera Gomez A, Sogin ML: **Benthic eukaryotic diversity in the Guaymas Basin hydrothermal vent environment**. *Proc Natl Acad Sci U S A* 2002, **99**(11):7658-7662.

7. Dawson SC, Pace NR: **Novel kingdom-level eukaryotic diversity in anoxic environments**. *Proc Natl Acad Sci U S A* 2002, **99**(12):8324-8329.

8. DeLong EF: **Archaea in coastal marine environments**. *Proc Natl Acad Sci U S A* 1992, **89**(12):5685-5689.

9. Sogin ML, Gunderson JH: **Structural diversity of eukaryotic small subunit ribosomal RNAs. Evolutionary implications**. *Ann N Y Acad Sci* 1987, **503**:125-139.

10. Amann RI, Binder BJ, Olson RJ, Chisholm SW, Devereux R, Stahl DA: **Combination of 16S rRNA-targeted oligonucleotide probes with flow cytometry for analyzing mixed microbial populations**. *Appl Environ Microbiol* 1990, **56**(6):1919-1925.

11. Medlin L, Elwood HJ, Stickel S, Sogin ML: **The characterization of enzymatically amplified eukaryotic 16S-like rRNA-coding regions**. *Gene* 1988, **71**(2):491-499.

12. Hadziavdic K, Lekang K, Lanzen A, Jonassen I, Thompson EM, Troedsson C: **Characterization of the 18S rRNA gene for designing universal eukaryote specific primers**. *PLoS One* 2014, **9**(2):e87624.

13. Chen Y, Chen Z, Guo R, Chen N, Lu H, Huang S, Wang J, Li L: **Correlation between gastrointestinal fungi and varying degrees of chronic hepatitis B virus infection**. *Diagn Microbiol Infect Dis* 2011, **70**(4):492-498.

14. He XL, Li Q, Peng WH, Zhou J, Cao XL, Wang D, Huang ZQ, Tan W, Li Y, Gan BC: **Intra- and inter-isolate variation of ribosomal and protein-coding genes in Pleurotus: implications for molecular identification and phylogeny on fungal groups**. *BMC Microbiol* 2017, **17**(1):139.

15. Vainio EJ, Hantula J: **Direct analysis of wood-inhabiting fungi using denaturing gradient gel electrophoresis of amplified ribosomal DNA**. *Mycological Research* 2000, **104**(8):927-936.

16. Bradley IM, Pinto AJ, Guest JS: **Design and Evaluation of Illumina MiSeq-Compatible, 18S rRNA Gene-Specific Primers for Improved Characterization of Mixed Phototrophic Communities**. *Appl Environ Microbiol* 2016, **82**(19):5878-5891.

17. Zhan A, Hulák M, Sylvester F, Huang X, Adebayo AA, Abbott CL, Adamowicz SJ, Heath DD, Cristescu ME, MacIsaac HJ: **High sensitivity of 454 pyrosequencing for detection of rare species in aquatic communities**. 2013, **4**(6):558-565.

18. White T, Bruns T, Lee S, Taylor J: **Amplification and direct sequencing of fungal ribosomal RNA genes for phylogenetics**. In: *PCR Protocols: A Guide to Methods and Applications.* Edited by Innis M, Gelfand D, Shinsky J, White T: Academic Press; 1990: 315-322.

19. Gardes M, Bruns TD: **ITS primers with enhanced specificity for basidiomycetes--application to the identification of mycorrhizae and rusts**. *Mol Ecol* 1993, **2**(2):113-118.

20. McLaughlin GL, S. S. Ssenyonga, E. Nanteza, Rubaire-Akiiki, O. Wafula, R. D. Hansen, M. H. Vodkin, R. J. Novak, V. R. Gordon, S. Montenegro- James, M. James, H. Aviles, R. Armijos, C. Santrich, K. Weigle, N. Saravia, E. Wozniak, O. Gaye, R. Mdachi, S. Z. Shapiro, K.-P. Chang, and I. Kakoma: **PCR-based detection and typing of parasites**. Wallingford, Oxon, United Kingdom: CAB International; 1996.

21. Chabchoub N, Abdelmalek R, Mellouli F, Kanoun F, Thellier M, Bouratbine A, Aoun K: **Genetic identification of intestinal microsporidia species in immunocompromised patients in Tunisia**. *Am J Trop Med Hyg* 2009, **80**(1):24-27.

22. Corbeil LB, Campero CM, Van Hoosear K, Bondurant RH: **Detection of trichomonad species in the reproductive tracts of breeding and virgin bulls**. *Vet Parasitol* 2008, **154**(3-4):226-232.
